# Fast disintegrating pellets: Formulation and evaluation

**DOI:** 10.12688/f1000research.165282.1

**Published:** 2025-07-21

**Authors:** Suhad Anabousi, Hani Naseef, Moammal Qurt, Abdallah AbuKhalil, Abdullah Rabba

**Affiliations:** 1 Department of Pharmacy, Faculty of Pharmacy, Nursing and Health Professions, Birzeit University, West Bank,, State of Palestine, 14, Palestinian Territory

**Keywords:** Pellets, Extrusion-spheronization, Fast disintegrating pellets, microcrystalline cellulose, super disintegrant, combination

## Abstract

**Background:**

Extrusion-spheronization is the most commonly used technology to produce pellets using microcrystalline cellulose as a pelletizing agent. However, it has the major drawbacks of lack of disintegration and prolonged drug release. This study aimed to develop rapidly disintegrating microcrystalline cellulose-based pellets.

**Methods:**

Several pellet formulations were prepared via extrusion spheronization using a combination of microcrystalline cellulose, mannitol, polyethylene glycol 400(PEG 400), polyplasdone (PPXL), and croscarmellose sodium (CCS). Subsequently, they were evaluated for their physical characteristics.

**Results:**

Process optimization indicated that 500 RPM is the ideal extrusion speed. Furthermore, the best spheronization speed was to start with a speed of 3000 RPM speed to cut off the extrudate at a shorter length and then lower the speed to 1000 RPM to reduce fine production and allow for spherical pellet formation. Increasing the polyethylene glycol content to 20% and maintaining the percentages of croscarmellose sodium (15%), 15%), and polyplasdone xl (5%), respectively, demonstrated a significant improvement in disintegration time (DT).

**Conclusions:**

MCC-based pellets with fast-disintegrating characteristics were obtained by extrusion and spheronization. Incorporating the soluble filler mannitol, hydrophilic polymer PEG 400 with super-disintegrant CCS, and PPXL 400 resulted in a more porous matrix that facilitated water entry and rapid swelling of the pellets to explode and disintegrate quickly (2 min).

**List of abbreviations**
(APIs): Active pharmaceutical ingredients
A: Area
AR: Aspect ratio
°C: Celsius
C: Circularity
CCS: Croscarmellose Sodium
D: Diameter
DT: Disintegration time
E-S
: Extrusion-spheronization
F. D: Ferret diameter
Gm: Gram
hrs: Hours
L.O.D: Loss on drying
MCC: Microcrystalline cellulose
μm: Micrometer
Mg: Milligram
Ml: Milliliter
min.: Minutes
MUPS: Multiunit particulates
P: Perimeter
PEG 400: Polyethylene glycol 400
PPXL: Polyplasdone XL10
PVP: Povidone
R: Radius
RN: Roundness
sec.: Second
S. load: Spheronization load
SQRT: Square root
w/w: Weight/weight
Ps. HCl: Pseudoephedrine hydrochloride
orph; citr.: orphenadrine citrate
RSD: Relative standard deviation

## 1. Introduction

The oral route remains the most favorable method for drug administration because of its convenience of application, ^1, 2^ pain avoidance, and reduced production costs. ^3^ Multiunit particulates (MUPS) include various dosage forms such as granules, pellets, and mini-tablets. ^4^ Compared with monolithic dosage forms, MUPS offers a variety of advantages, including less reliance on gastrointestinal emptying, which results in less inter-and intra-subject variation in gastrointestinal residence time and a lower likelihood of localized adverse effects. ^5^ Pellets are small spherical or semi-spherical multi-particulates with a mean diameter of 0.5 to 2 mm, consisting of fine powder of excipients and active pharmaceutical ingredient (API). ^6^ Pellets are the most attractive form due to their several technological and pharmacological advantages, such as free flowability, even size distribution, reduced risk of dose dumping, and ability to combine many incompatible drugs, as well as different release profiles in one dosage form, which helps elderly patients by reducing the number of daily doses. ^7– 9^ Pellets can be formed using various technologies based on various principles. However, extrusion-spheronization (ES) and layering technologies are the most commonly used pelletization processes. ^7^ Extrusion spheronization is a technique used to produce pellets appropriate for immediate and controlled-release dosage forms. ^10^ ES is a two-stage process in which a soft solid material is created by combining the excipient, active pharmaceutical ingredients (APIs), and binder liquid, which is then extruded to produce rods of a specific diameter and spheronized into spherical, dense pellets that are dried or processed. ^11^

Microcrystalline cellulose (MCC) is a biopolymer generated from wood pulp and used as an excipient in the manufacture of pharmaceutical tablets and capsules. ^11^ It has various grades and sizes. MCC - PH 101 is most commonly used for ES. ^12^ MCC is the most attractive pelletization excipient employed in the extrusion/spheronization process for developing pellets for pharmaceutical purposes. It has superior water uptake capacity, water-holding ability, ideal rheological qualities, plasticity, and cohesiveness. ^13^

Many Strategies to rapidly disintegrate MCC-based pellets have been used, such as promoting pellet disintegration by incorporating super-disintegrants and adding soluble fillers, ^13^ increasing pellet porosity by changing the granulating liquid, modulating drying conditions, and incorporating pore formers. ^13^ In addition, the partial substitution of MCC by the soluble filler retains the advantages of MCC while adding the functional quality provided by the additional components. ^13^

Kunam et al. used crospovidone to produce fast disintegrating pellets and noticed that it increased the dissolution of Ezetimibe by 1-2 fold compared to the marketed conventional dosage forms. ^14^ Souto C and co-workers studied the effects of croscarmellose sodium (CCS) and sodium starch glycolate on the dissolution rate of pellets containing hydrochlorothiazide. However, only a slight increase in drug release has been observed. ^15^ Goyanes studied the use of mannitol in hydrochlorothiazide pellets and observed that mannitol had a satisfactory effect on pellet morphology and enhanced drug release because of its high solubility and ability to create pores in pellets when dissolved. ^16^ The concentration of mannitol substantially increases the drug dissolution rate from pellets, producing small pellets. ^5^ Shah et al. observed that pellets made with a 40% 2-propanol/water mixture granulating liquid exhibited a faster dissolution rate than those made with a lower proportion of 2-propanol. This is due to the rapid and complete disintegration of the pellets. The pellet strength decreased, and a less uniform shape was produced as the 2-propranolol level in the ethanol/water fluid increased owing to an alteration in the particle bonding of the pellets. ^17^ Chamsai et al. studied the effects of polyethylene glycol (PEG) 400, croscarmellose sodium, and polysorbate 80 with MCC and granulated them with an ethanol solution to achieve fast disintegration of indomethacin. ^18^ Vervaet noticed that using polyethylene glycol 400 and hydrogenated castor oil enhanced the release rate of hydrochlorothiazide from MCC PH 101 pellets. ^19^ Kranz et al. studied preparing pellets with a high drug loading of 90% and immediate release properties using only a small quantity of super disintegrant and pore former PEG 6000. ^20^ C. Vervaet found that MCC can tolerate up to 43% (w/w) of PEG 400 and will be free-flowing. At higher concentrations, pellets were attached to each other. In addition, he noticed that solubilizing PEG 400 is a promising excipient to enhance the dissolution of poorly soluble drugs. ^19^ Afrasiabi et al. used CCS in conjunction with PEG and found that it had a significant impact on increasing the dissolution rate, which is attributed to the increased pores in the inert matrix caused by the presence of soluble PEG and the increased surface area of pellets, in addition to the presence of disintegrants. ^21^

Despite its excellent properties, drawbacks related to the use of MCC have been reported. The most common disadvantage is a delayed or inadequate drug release profile caused by a lack of disintegration, as the pellet shrinks significantly during the drying process, specifically when used in high doses with a poorly soluble medication. This property restricts the use of MCC in immediate-release dosage forms. Therefore, this study aimed to prepare fast disintegrating pellets using the soluble filler mannitol, pore former polyethylene glycol 400, and the super disintegrant combination croscarmellose sodium and polyplasdone XL10 for the synergistic effect of altered disintegration properties of MCC-based pellets.

## 2. Materials and methods

### 2.1 Materials

Microcrystalline cellulose (MCC) PH 101(Lot. No. 245324571), croscarmellose sodium (CCS) (Lot. No. 201803278), mannitol (Lot. No. 773672023), polyplasdone xl 10 (PPXL) (Lot. No. RN537), polyvinylpyrrolidone (PVP K30), polyethylene glycol (PEG) 400 (Lot. No. YY00I2R501), pseudoephedrine hydrochloride (Lot. No. 201907079), and orphenadrine citrate (Lot. No. 9202003001). All materials and reagents were of pharmaceutical grade and donated by Jerusalem Pharmaceuticals Co., Ltd. (Palestine).

### 2.2 Extrusion-spheronization process

Extrusion-spheronization was used to prepare several pellet formulations containing a mixture of various excipients, with and without active ingredients, using a local multi-lab device. The ingredients for each formula were mixed using a laboratory-size mixer, according to the compositions listed in Table 1. First, multiple trials were conducted to determine the appropriate amount of binder liquid based on extrusion ability and pellet quality. Then, PVP was dissolved in distilled water in a beaker, PEG400 was added, and the solution was used to moisten the dry mixture. Subsequently, 5 ml of the binder solution was added every 30 s during constant mixing, and the process was continued until the desired plastic mass was obtained. The resulting wet mass was extruded at a speed of a screw extruder ranging from 300 to 1000 RPM through a screen with a 1 mm die diameter. Approximately 15 g of the extrudate was loaded into a spheronizer with a cross-hatched friction plate. Spheronization was performed on the extrudates at speeds ranging from 1000 to 5000 rpm until spherical pellets were produced. The pellets were dried for 6 h in a tray dryer at 50°C. Finally, a sieve shaker was used to separate pellets with a size fraction of 600-850 μm (Retsch, Germany). Pellets were maintained at room temperature in sealed glass vials for evaluation.

**Table 1.** Composition of the pellet formulations.

	MCC (%)	PP XL (%)	CCS (%)	Mannitol (%)	PEG 400 (%)	PVP (%)	Ethanol	Ps. HCl%	Orph. Citr.%	Water
X1	70.6	--	--	14.4	14.4	0.6	--	--	--	Q.s
X2	60	--	--	25	14.4	0.6	--	--	--	Q.s
X3	51	--	--	34	14.4	0.6	--	--	--	Q.s
X4	65	--	--	14.4	20	0.6	--	--	--	Q.s
P1	47	4	--	34	14.4	0.6	--	--	--	Q.s
P2	43	8	--	34	14.4	0.6	--	--	--	Q.s
P3	33.33	16.67	--	35	14.4	0.6	--	--	--	Q.s
P4	25	25	--	35	14.4	0.6	--	--	--	Q.s
P5	16.67	33.33	--	35	14.4	0.6	--	--	--	Q.s
PE1	43	8	--	34	14.4	0.6	50% v/v	--	--	Q.s
C1	45	--	5	35	14.4	0.6	--	--	--	Q.s
C2	40	--	10	35	14.4	0.6	--	--	--	Q.s
C3	35	--	15	35	14.4	0.6	--	--	--	Q.s
C4	25	--	25	35	14.4	0.6	--	--	--	Q.s
CP1	45	2.5	2.5	35	14.4	0.6	--	--	--	Q.s
CP2	40	5	5	35	14.4	0.6	--	--	--	Q.s
CP3	35	5	10	35	14.4	0.6	--	--	--	Q.s
CP4	30	5	15	35	14.4	0.6	--	--	--	Q.s
CP5	30	10	10	35	14.4	0.6	--	--	--	Q.s
CP6	50	5	5	25	14.4	0.6	--	--	--	Q.s
CP7	45	5	10	25	14.4	0.6	--	--	--	Q.s
CP8	40	10	10	25	14.4	0.6	--	--	--	Q.s
CP9	40	15	15	15	14.4	0.6	--	--	--	Q.s
CP10	45	15	15	10	14.4	0.6	--	--	--	Q.s
CP11	40	15	15	9.4	20	0.6	--	--	--	Q.s
CP12	50	5	15	9.4	20	0.6	--	--	--	Q.s
CPP1	50	5	15	9.4	20	0.6		+5%	--	Q.s
CPP2	50	5	15	9.4	20	0.6		+15%	--	Q.s
CPP3	50	5	15	9.4	20	0.6		+25%	--	Q.s
CPP4	50	5	15	9.4	20	0.6		+30%	--	Q.s
CPO1	50	5	15	9.4	20	0.6		--	+5%	Q.s
CPO2	50	5	15	9.4	20	0.6		--	+15%	Q.s
CPO3	50	5	15	9.4	20	0.6		--	+25%	Q.s
CPO4	50	5	15	9.4	20	0.6		--	+30%	Q.s

Microcrystalline cellulose (MCC) PH 101, croscarmellose sodium (CCS), polyplasdone xl 10 (PPXL), polyvinylpyrrolidone (PVP K30), polyethelenglycol (PEG) 400, Ps. HCl: Pseudoephedrine hydrochloride, orph, citr.: Orphenadrine citrate.

The two model drugs, pseudoephedrine hydrochloride and orphenadrine citrate, were uploaded to the final formulas 5%, 15%, 25%, and 30% separately. The two model drugs have different degrees of solubility: pseudoephedrine hydrochloride is freely soluble in water, and orphenadrine citrate is sparingly soluble in water.

### 2.3 Pellets evaluation

***2.3.1 Disintegration test***

A USP tablet disintegration apparatus was used to study the pellet disintegration. First, a 300 μm mesh was placed at the bottom of each tube in the basket-rack assembly to prevent the pellets from escaping. Next, 100 mg of pellets was placed in each of the six tubes of the basket rack assembly tubes, using water at 37± 2°C as the immersion fluid and reducing the fluid volume in the beaker from 800 ml to 700 ml to ensure that pellets remained in the tube. The time the pellets passed through the 300 μ mesh was recorded as the DT.

***2.3.2 Particle size shape and size analysis***

The particle size of the pellet was determined by sieve shaking. First, by arranging a group of sieves with different aperture sizes in descending order (1.18 mm, 850, 600, 425, 250 μm) using a sieve shaker (Retsch AS200, Germany) for 5 min, the weight portion kept on each sieve was weighed using an analytical balance (Adam, USA). Each fraction percentage was then calculated, and the fraction size range of 600-850 μm was used for further investigation.

The size and shape of the pellets were evaluated using a USB digital microscope (China) connected to a computer by capturing photos of the pellets. The license-free image analysis software ImageJ ^®^ was used to analyze the images. The magnification was set such that each pixel was 0.0866 μm. Approximately 100 pellets from the to 600-850 μm size fraction of each batch were examined to determine the projected area, perimeter, Feret diameter (mean of 180 caliper measurements with a 1° rotation angle), circularity, aspect ratio (AR) (the ratio of the longest Feret diameter to its longest perpendicular diameter), roundness, solidity, and sphericity for each pellet.

***2.3.3 Moisture content***

The pellets were crushed with a mortar and pestle, and the loss on drying (LOD) was determined by heating approximately 5 g of precisely weighed samples on a sample pan using a moisture analyzer (OHAUS, Switzerland).

***2.3.4 Friability***

A sample of 11.6 gm of pellets was weighed and placed in a friability tester drum with 200 glass beads with a diameter of 4 mm, and the device was rotated at 100 rpm for 4 min. The pellets were sieved for 5 min. by 250 μm mesh to remove fines, and the weight was noted. Then, friability was calculated as follows:

$$\text{friabilty}=\frac{W1-W2}{W1}*100\%$$

W1 and W2 are the initial and final weights of the pellets, respectively.

***2.3.5 Camera capture of the pellet disintegration process***

In addition to the disintegration endpoint studied using the USP disintegration apparatus, the pellets disintegrated into particles of various sizes when evaluated at a static position. A few drops of water were placed on the pellets on the opaque surface. A USB digital microscope (China) was connected to a computer to capture the disintegration process. Images were captured from the beginning until the pellet disintegrated or exploded into small fragments. Pellet images were acquired every 30 seconds. for formulations containing polyplasdone XL 10 and croscarmellose sodium.

## 3. Results and discussion

### 3.1 Formulation development

Many operational variables can influence the pellet characteristics during the extrusion, spheronization, and drying stages. For example, the extruder speed, screen thickness, opening diameter, friction plate type, spheronization time, speed, load, drying temperature, and time are all variables that determine the final pellet quality. In addition, formulation variables such as the addition of a binder, filler, disintegrant, and type and quantity of granulating liquid would also affect the final pellet quality. Therefore, the success of the method can be described as being formulation-dependent. ^22^

The results revealed that MCC, as a spheronizing aid, significantly impacts sphericity when combined with a granulating solvent such as water, which functions as a plasticizer. Furthermore, MCC slows pellet disintegration and affects the DT. ^3^ In this study, several pellets were prepared and analyzed (without disintegrant, with one disintegrant, and with a combination of disintegrant and active substance). Furthermore, the impact of different process parameters on the pellet quality was investigated.

### 3.2 Development of pellets without disintegrant (X1-X4)

The development process began with the preparation and evaluation of pellet formulations without disintegrants **.** Formulations X1 to X4 ( Table 1) were prepared using MCC PH 101 as a pelletization aid to evaluate the possibility of pellet formation. The PVP quantity (0.6%) was determined based on early trials, with the formation of a minimum proportion of fines during spheronization. Initial studies were undertaken with various percentages of the soluble filler mannitol added to MCC, granulated with different amounts of aqueous PVP binder solution, and PEG 400 until a soft wet mass was achieved. The resulting wet mass was then extruded at a constant speed (500 RPM speed) using a screw extruder, and the extrudates were spheronized and dried. Disintegration and shape of the pellets were assessed ( Table 2). All formulations (X1 to X4) showed an excellent spherical shape, as evidenced by a pellet roundness of > 0.92, which was close to 1 ( Table 2). In addition, a high percentage of MCC was robust to the formula and allowed different time intervals for spheronization without affecting the final pellet shape.

**Table 2.** Results of disintegration test and pellets shape.

Formula #	S. time	S. load	speed	Microscopic image	Roundness			Pass/fail	DT	Pass/fail
					Pellet #					
					1	2	3			
X1	90 sec.	15 gm	3000 RPM	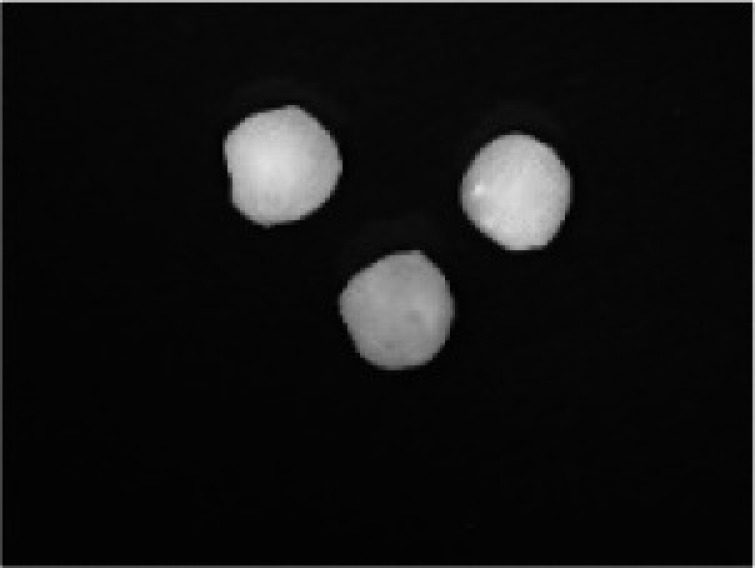	0.971	0.942	0.955	Pass	> 2 hrs	Fail
X2	60 sec.	15 gm	3000 RPM	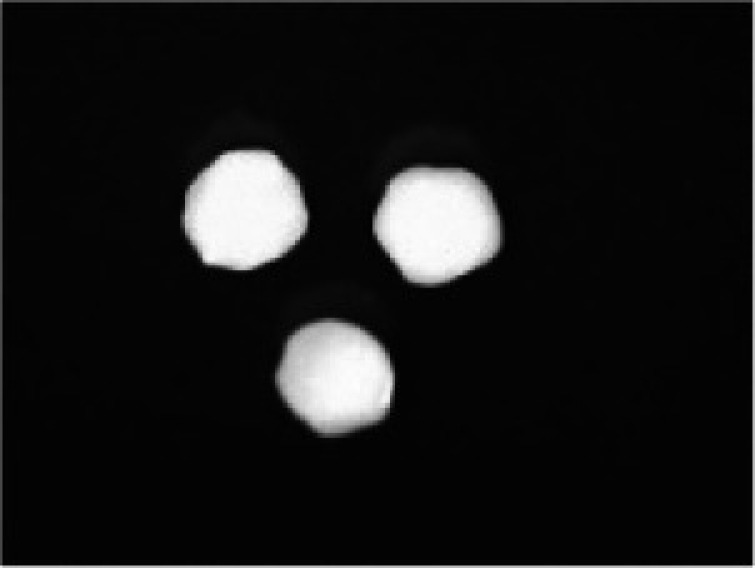	0.940	0.940	0.920	Pass	> 2 hrs	Fail
X3	90 sec.	15 gm	3000 RPM	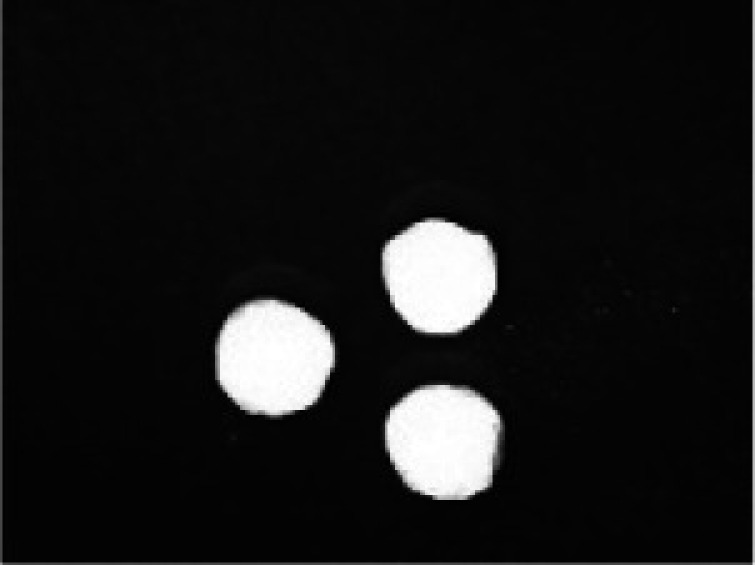	0.963	0.964	0.950	Pass	> 2 hrs	Fail
X4	120 sec.	15 gm	3000 RPM	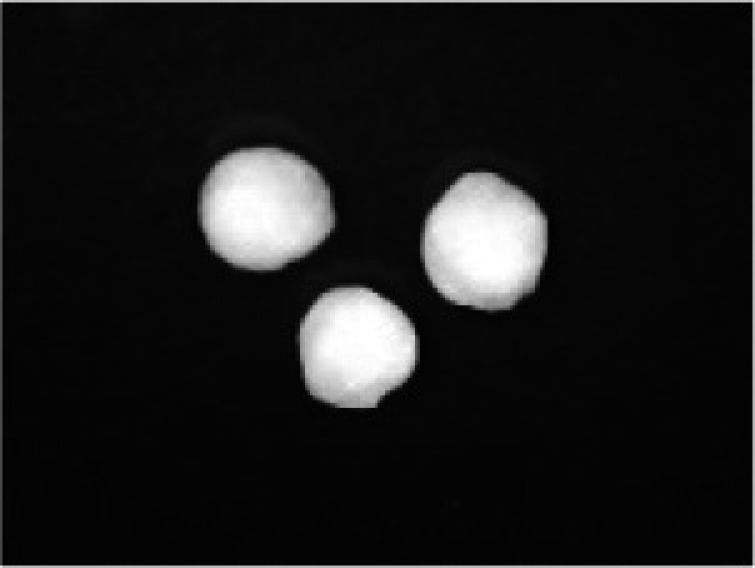	0.928	0.931	0.920	Pass	> 2 hrs	Fail
P1	30 sec.	15 gm	3000 RPM	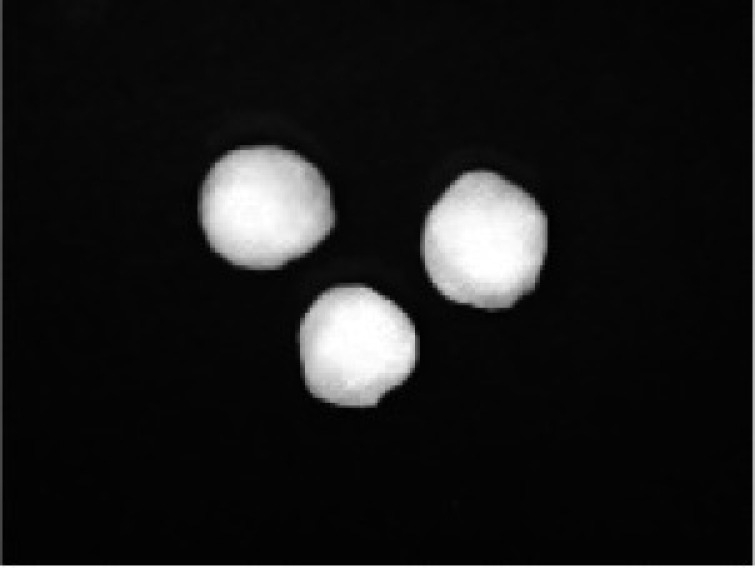	0.975	0.946	0.939	Pass	> 2 hrs	Fail
P2	30 sec.	15 gm	3000 RPM	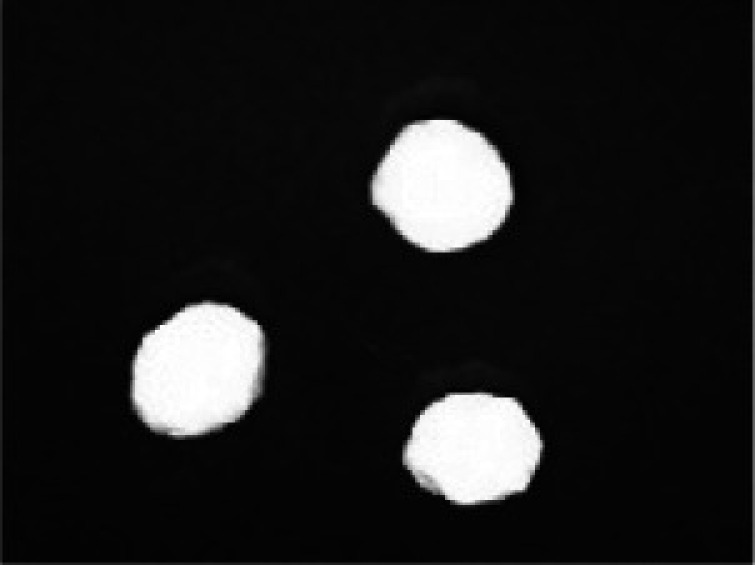	0.932	0.828	0.821	Pass	> 2 hrs	Fail
P3	30 sec.	15 gm	3000 RPM	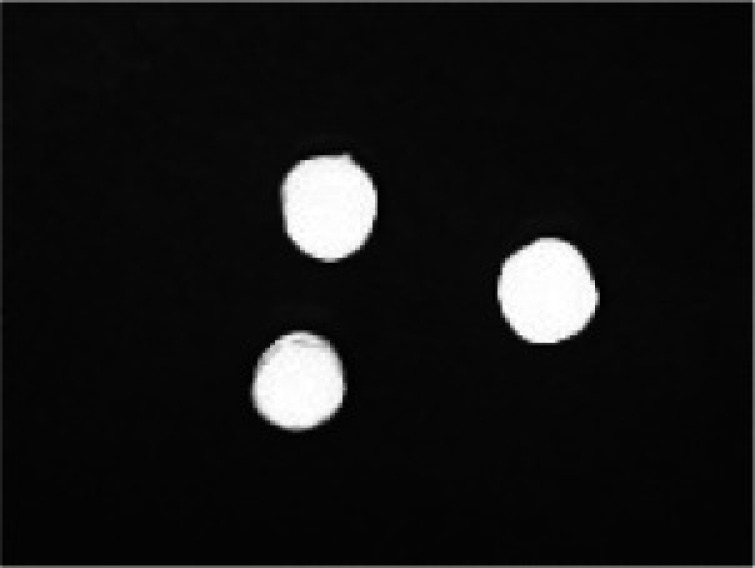	0.910	0.945	0.914	Pass	> 30 min	Fail
P4	30 sec.	15 gm	3000 RPM	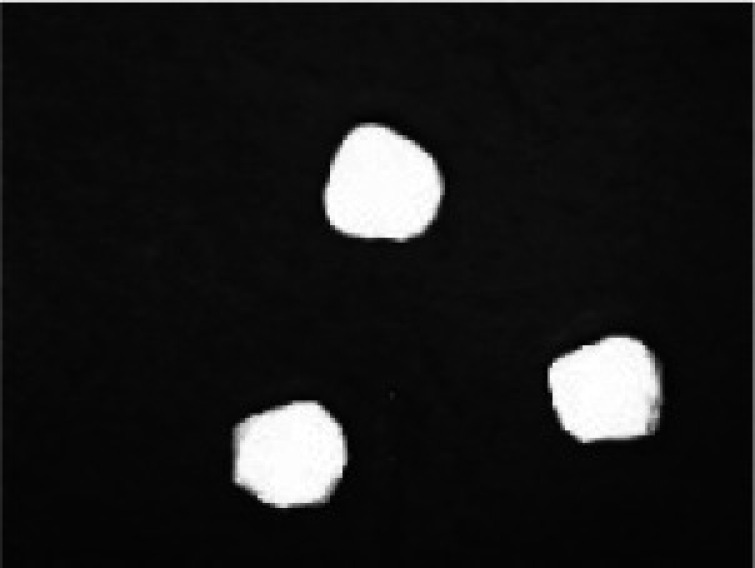	0.916	0.918	0.902	Pass	> 30 min.	Fail
P5	30 sec.	15 gm	3000 RPM	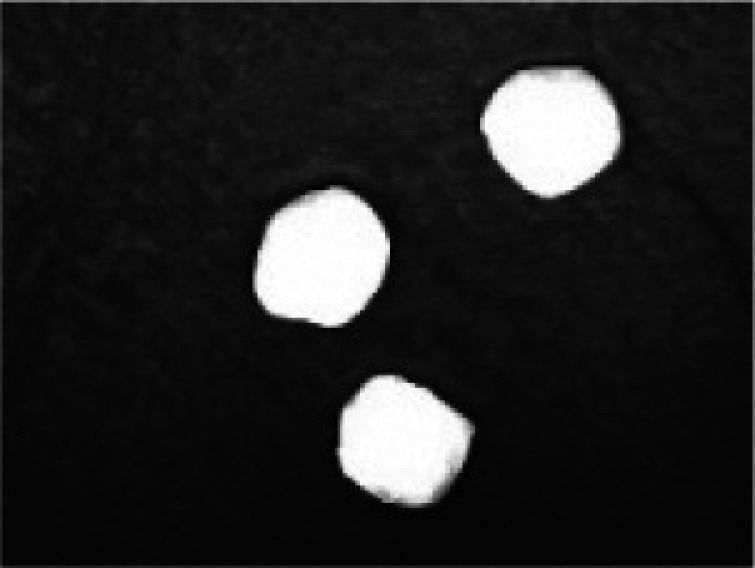	0.942	0.883	0.911	Pass	> 30 min.	Fail
C1	30 sec.	15 gm	3000 RPM	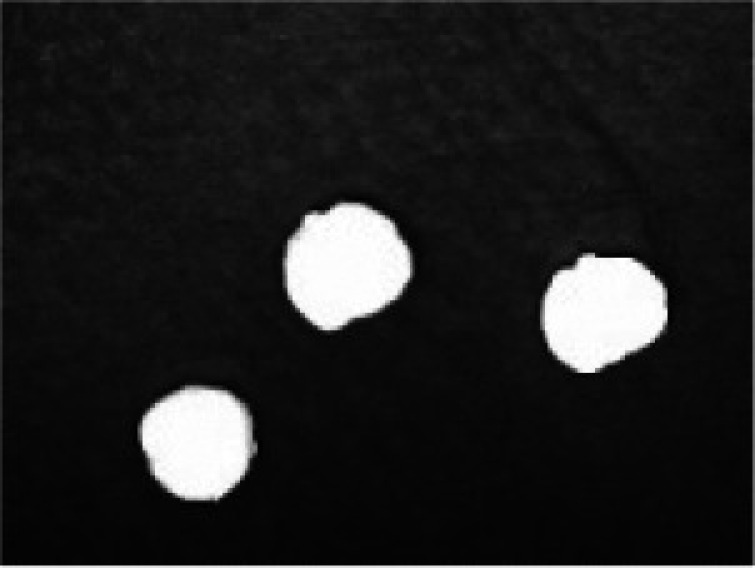	0.909	0.849	0.996	Pass	> 30 min.	Fail
C2	30 sec.	15 gm	3000 RPM	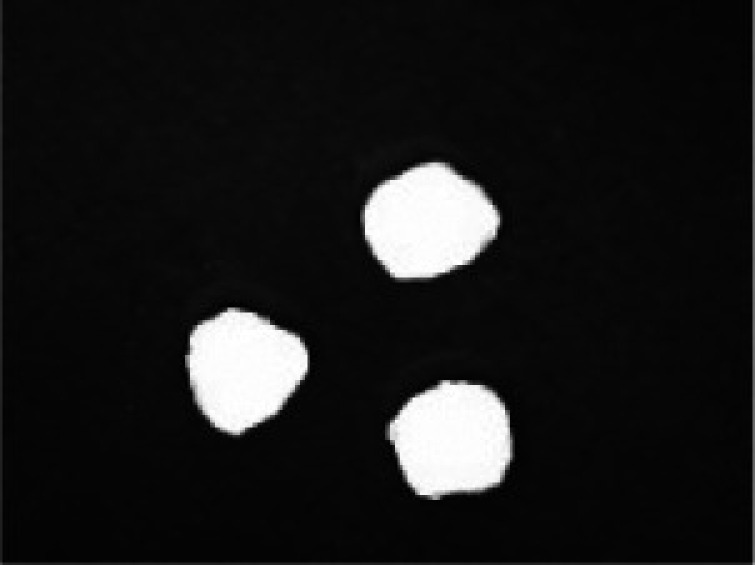	0.838	0.968	0.942	Pass.	> 30 min.	Fail
C3	30 sec.	15 gm	3000 RPM	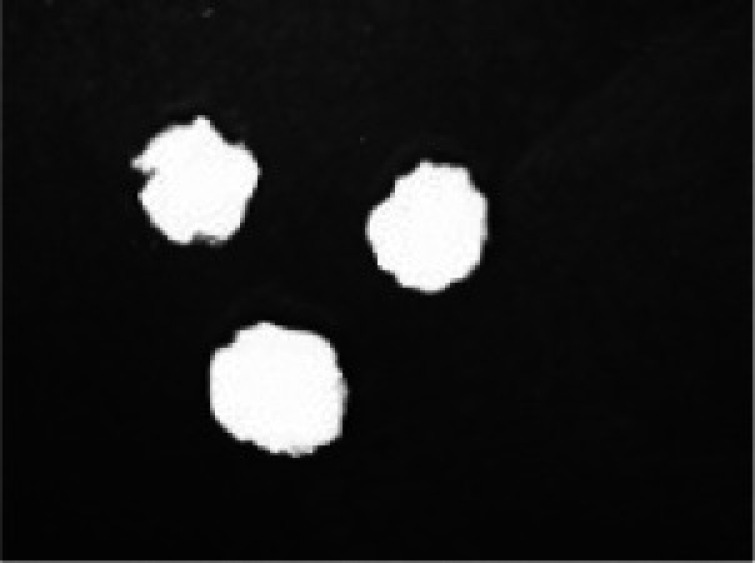	0.946	0.893	0.888	Fail	> 30 min.	Fail
C4	30 sec.	15 gm	3000 RPM	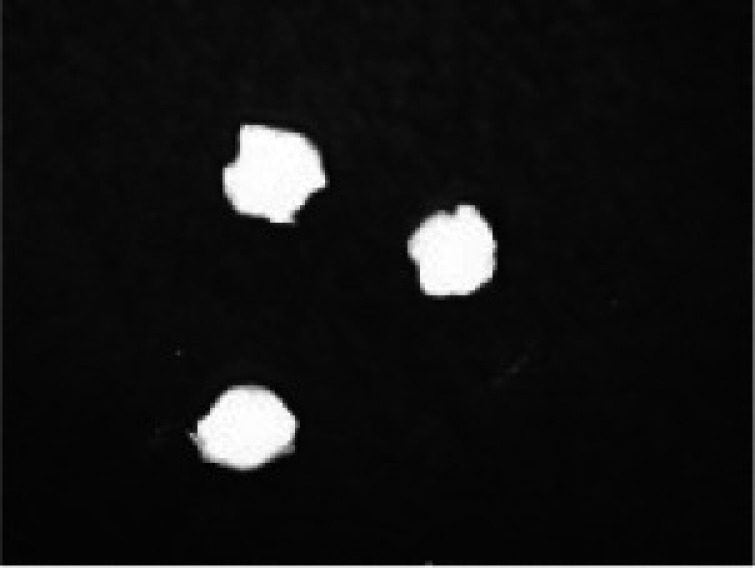	0.923	0.938	0.810	Fail	< 2 min.	Pass
CP1	30 sec.	15 gm	3000 RPM	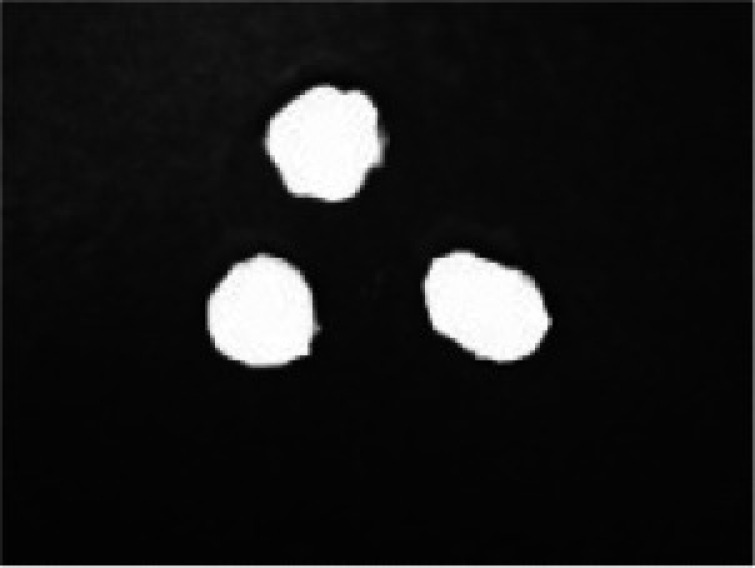	0.956	0.706	0.977	Fail	> 30 min.	Fail
CP2	30 sec.	15 gm	3000 RPM	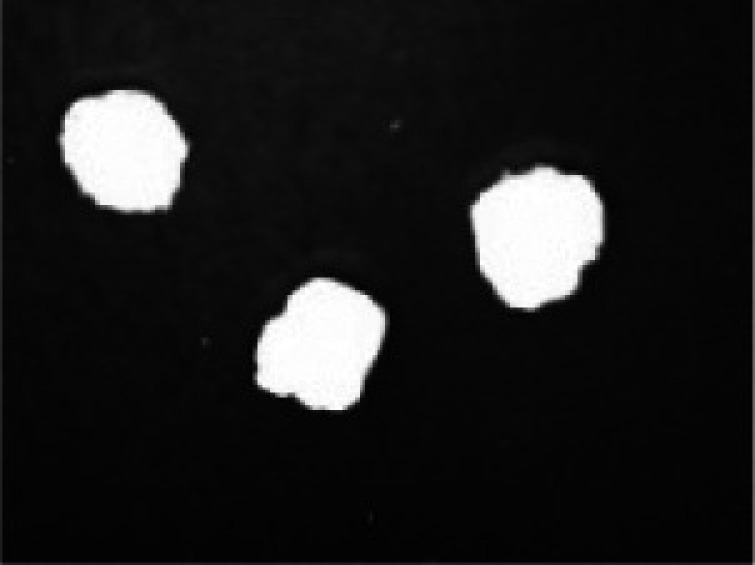	0.872	0.897	0.854	Fail	> 30 min.	Fail
CP3	30 sec.	15 gm	3000 RPM	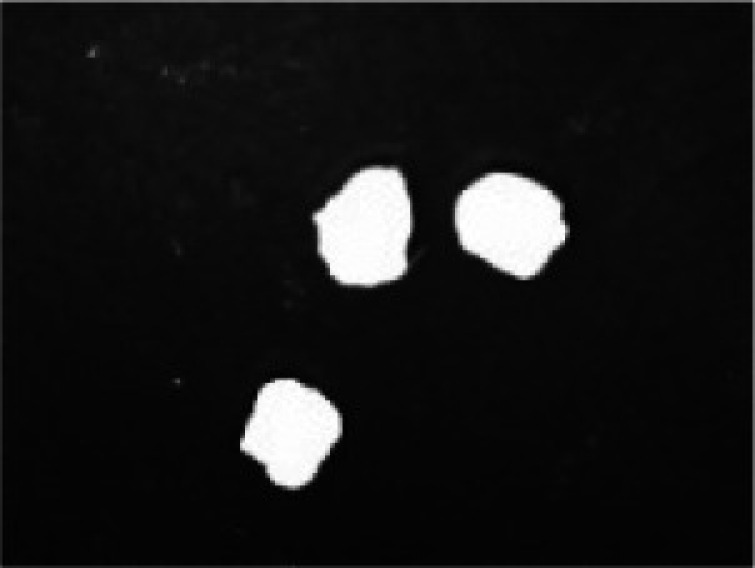	0.857	0.883	0.926	Fail	> 30 min.	Fail
CP4	30 sec.	15 gm	3000 RPM	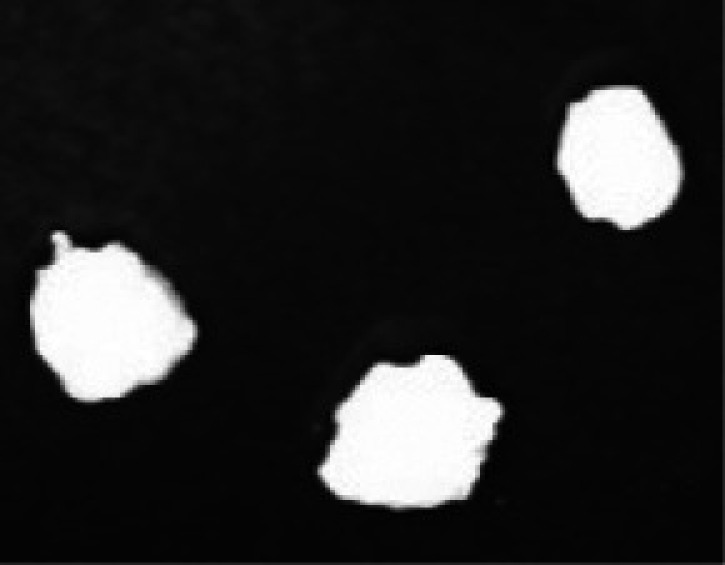	0.829	0.887	0.823	Fail	< 2 min.	Pass
CP5	30 sec.	15 gm	3000 RPM	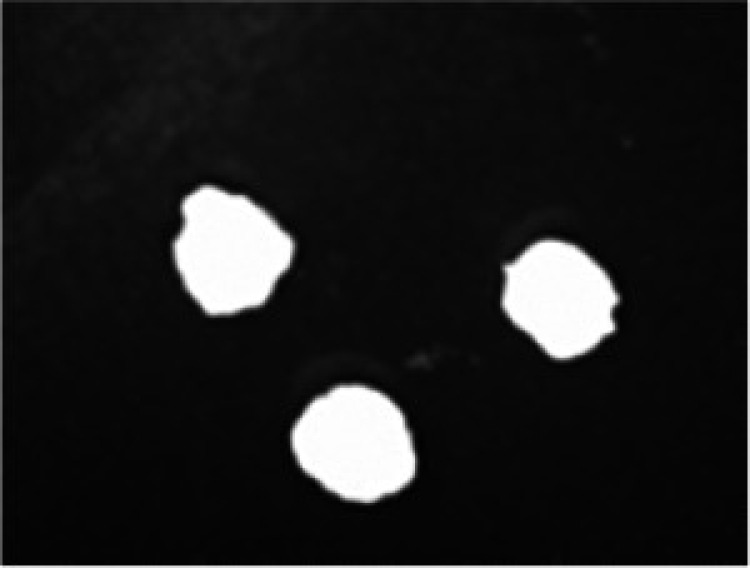	0.889	0.850	0.872	Fail	> 30 min.	Fail
CP6	30 sec.	15 gm	3000 RPM	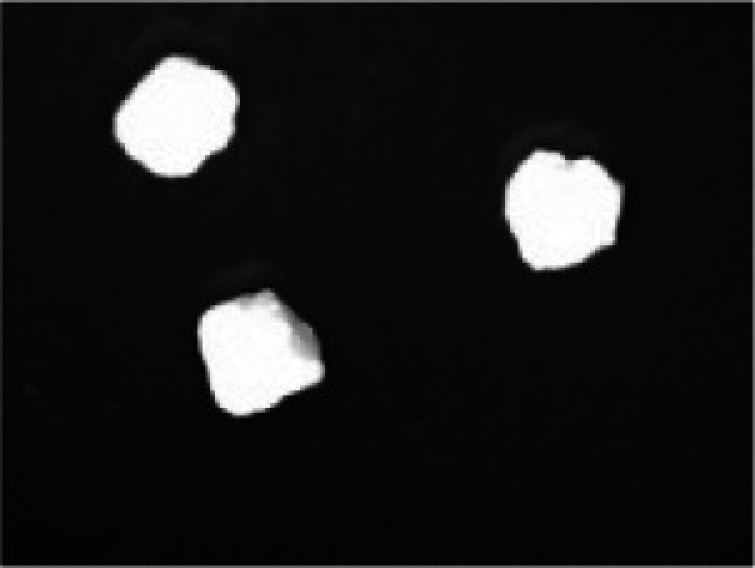	0.859	0.990	0.952	Fail	> 30 min.	Fail
CP7	30 sec.	15 gm	3000 RPM	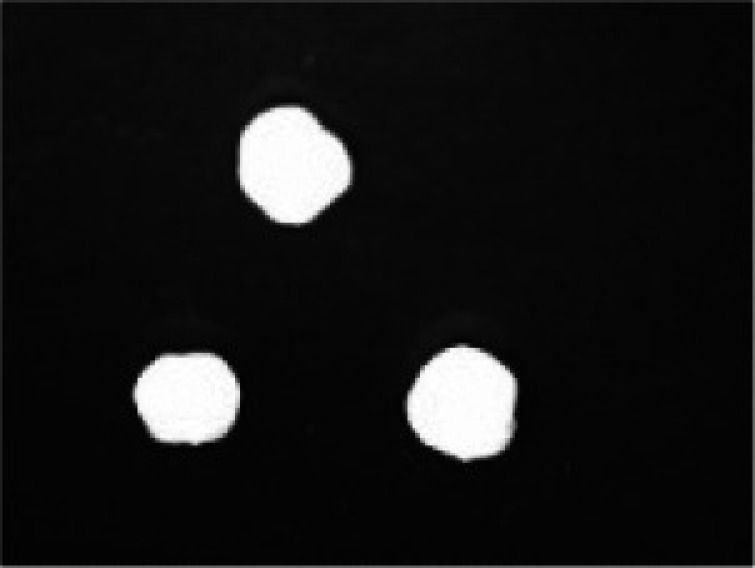	0.915	0.966	0.888	Fail	> 30 min.	Fail
CP8	30 sec.	15 gm	3000 RPM	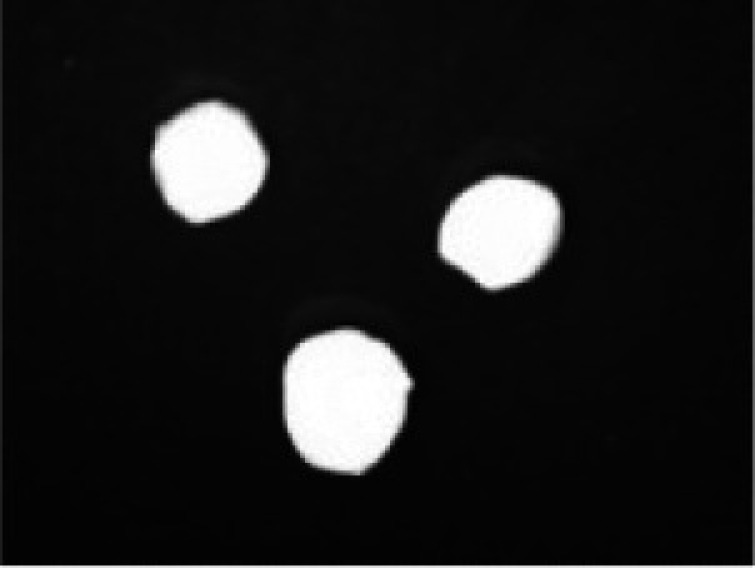	0.952	0.867	0.882	Fail	> 30 min.	Fail
CP9	30 sec.	15 gm	1000 RPM	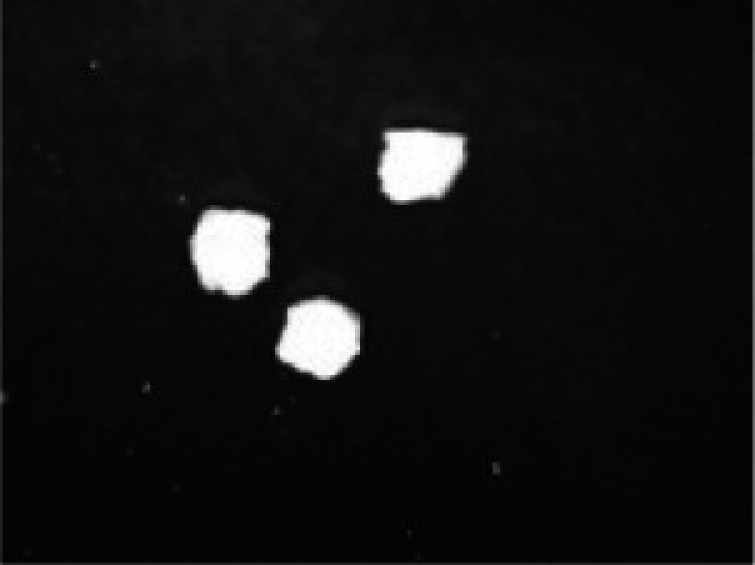	0.816	0.924	0.940	Fail	< 30 min.	Pass
CP10	30 sec.	15 gm	1000 RPM	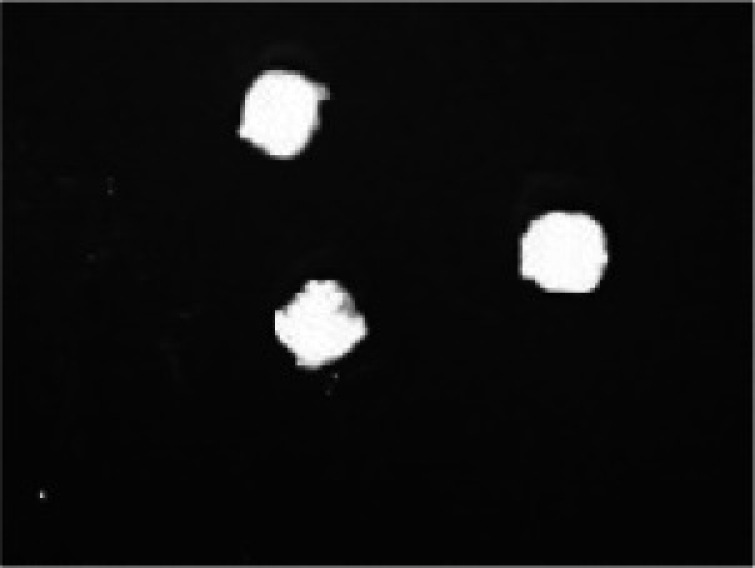	0.894	0.926	0.932	Fail	< 30 min.	Pass
CP11	30 sec.	15 gm	1000 RPM	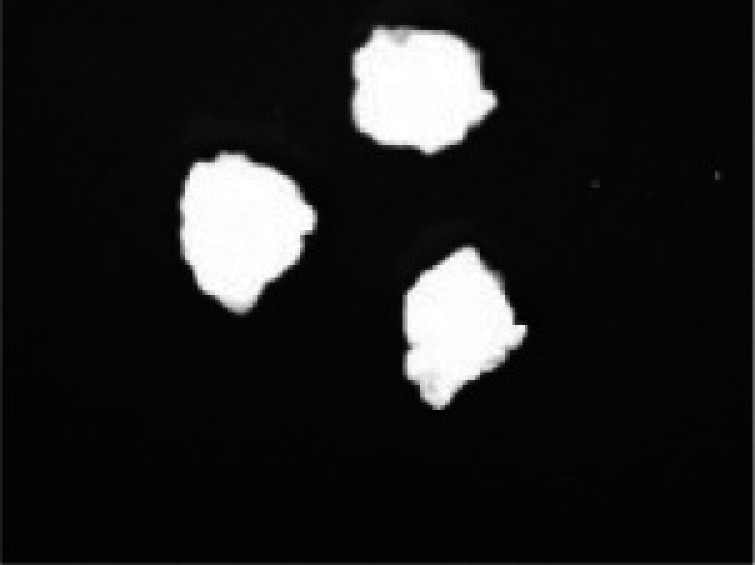	0.880	0.871	0.756	Fail	< 2 min.	Pass
CP12	30 sec.	15 gm	3000 RPM	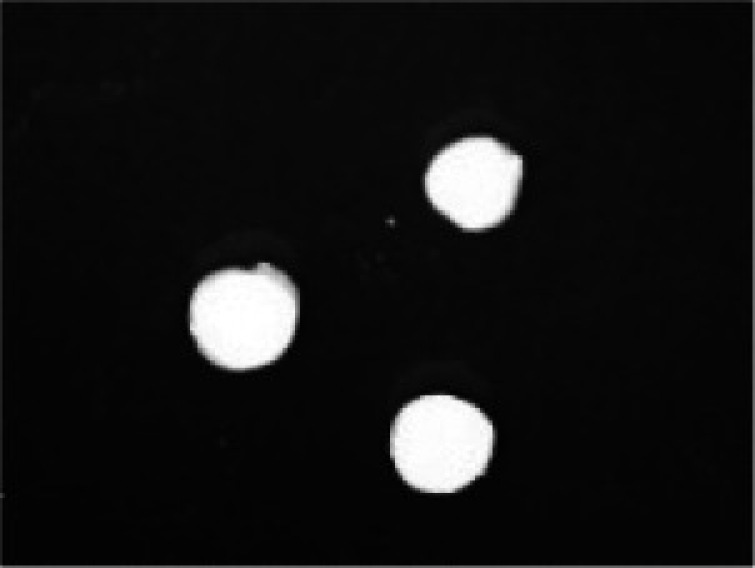	0.947	0.915	0.949	Pass	< 2 min.	Pass
CPP4	30 sec.	15 gm	3000 RPM	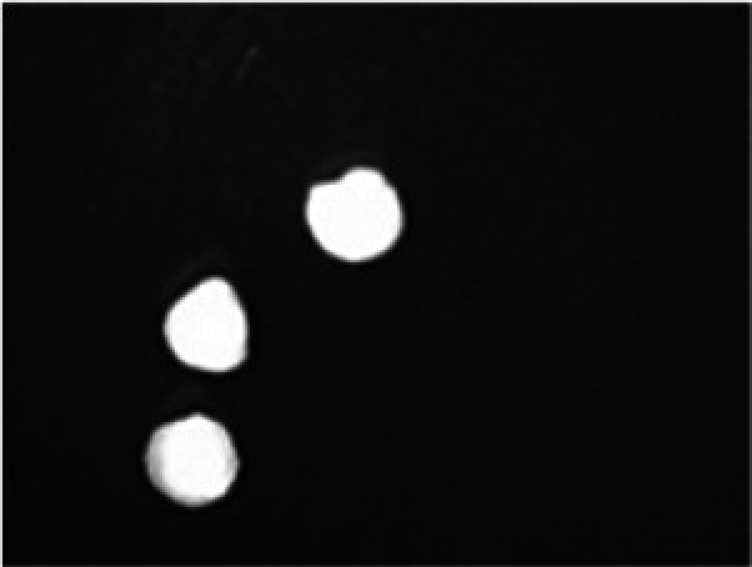	0.935	0.908	0.961	Pass	< 5 min.	Pass
CPO4	30 sec.	15 gm	3000 RPM	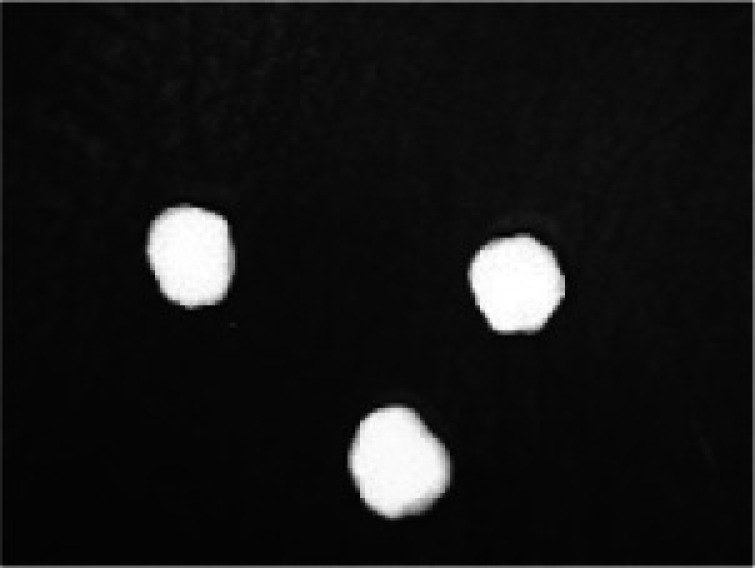	0.853	0.948	0.874	Pass	< 8 min.	Pass

S. time: Spheronization time, S. load: Spheronization load, DT: Disintegration time, min.: Minute.

However, a high MCC percentage retarded pellet DT owing to the high shrinkage of its structure during drying, which prevented water entry into the pellets. ^17, 23^

As illustrated in (X1 to X4, Table 2), MCC pellets containing mannitol and PEG did not disintegrate after 2 h, even when the mannitol content was increased to 34% and the PEG 400 was increased to 20%.

The results of (X1-X4) revealed that the optimal water amount was 1:1 of the MCC weight. A linear relationship between water content and MCC fraction was observed. The amount of water required for successful extrusion increased with the percentage of MCC in the formulation. Utilizing a small amount of water in the wet massing stage led to the production of less cohesive, brittle extrudates that are more prone to being destroyed by the rotating plate resulting in either higher production of fines or the formation of an extrudate that, despite its length reduction, remains cylindrical or “dumbbell” shaped. In addition, when a large amount of water is used, sticky dough is produced, which forms significantly larger pellets under centrifugal force. ^24^

### 3.3 Optimization of the extrusion-spheronization process

Because of its low cost and ability to produce high-quality pellets, extrusion-spheronization is the most extensively used pelletization technology. This method has several crucial factors that greatly influence the pellet properties. Extrusion pressure and speed, spheronization speed, pressure load, and duration are among these parameters. ^25^ To assess the impact of the variables in the current study, the percentages of different excipients used in formulations X1 to X4 were correlated with extrusion speed, spheronization load, speed, and time as variables to investigate the effects of mannitol and PEG 400 on the properties of MCC PH 101 pellets.

***3.3.1 Screw extruder speed***

The impact of screw extruder speed on the extrudate properties was investigated. At speeds ranging from 300 to 1000 RPM, the moist masses of the various formulations (X1 to X4) were extruded. The results revealed that 500 RPM was the optimal extrusion speed. Low speed produced less cohesive, brittle extrudates that broke apart early in the spheronization process, whereas high speed (1000 RPM) produced extrudates with surface defects, such as roughness and “shark skinning,” resulting in lower-quality pellets.

***3.3.2 Spheronization load time and speed***

The process of identifying the most suitable load was conducted by dividing the extrudate produced in each batch from formulations X1–X4 into three samples of approximately 10, 15, and 20 g. Each sample was spheronized at a fixed speed and time (3000 RPM for 30 s). The plate must be loaded correctly for the extrudate to be “chopped” and for the fragments to travel in a toroidal motion. The results showed that the appropriate weight of spheronizer to produce more spherical pellets was 15 g. The particle–particle interaction was insufficient when using a plate load of 10 g, and tiny irregular pellets were produced. When 20 g was used, the particles could not freely contact the spheronizer plate, resulting in the long-term production of spherical particles. ^24^

The impact of the spheronization speed on the final shape of the pellets was investigated by dividing the extrudate produced into three samples of approximately 15 g spheronized at different speeds and fixed times of 30 s. Consequently, the device offers a range of spheronization speeds: (1000-5000 RPM).

The findings showed that the best spheronization speed was to start with a speed of 3000 RPM speed to cut off the extrudate at a shorter length and then lower the speed to 1000 RPM to reduce fine production and allow for spherical pellet formation. The greatest variability in shape and size characteristics was observed at low speeds. This is because the extrudate broke down into smaller particles at this speed. However, because of the low energy input, the plastic deformation of the cylinder was not always complete, as the particle/particle and particle/spheronizer interactions were insufficient, and bone-shaped particles were produced. At the same time, the extrudate broke up at high speed, and a high percentage of fines was produced. ^26^

The extrudate produced in each batch was divided into five samples of approximately 15 g each, spheronized at 3000 RPM speeds and 1000 RPM speeds for 10, 30, 60, 90, and 120 s, respectively.

According to these findings, a short spheronization time (approximately 30 s) is sufficient to obtain pellets with the highest yield and adequate sphericity. Lower spheronization time (10 seconds) led to the production of irregular and rough pellets because the particles did not have enough time to round off, so “bone” shaped particles were produced. Longer spheronization periods (> 120 s) did not increase pellet sphericity but promoted pellet agglomeration and widening of the pellet size distribution. ^26^

As per the previous results, the most spherical pellets were achieved at a spheronization load of 15 g and spheronization speed of 3000 RPM, then 1000 RPM, and 30 s.

Referring to the above studies, pellets were produced at the optimum process parameters, and pellet sphericity and size distribution were evaluated visually and microscopically.

### 3.4 Development of pellets with one disintegrant (P1-P5 & C1- C5)

To enhance pellet disintegration, super-disintegrants were added, and an adequate amount of MCC was utilized to maintain the sphericity of the pellets. Several formulations (P1–P5 and C1–C4, Table 1) were produced using PPXL and CCS to evaluate the effect of the super-disintegrant.

Based on these results, sphericity is negatively affected by CCS. The pellet sphericity reached a maximum distortion at (15% and 25%) (C3 & C 4) of CCS content. In comparison, PPXL had no direct influence. All formulations showed a good spherical shape, as evidenced by the pellet roundness > 0.82, which is close to 1, except for C3 and C4, which have an apparent roughness and distortion of the surface to a wide size distribution ( Table 2).

The results of pellet evaluation ( Table 2) for the DT of different formulations showed that formulations P1 and P2, with (4% and 8%) PPXL content, respectively, had no significant effect on DT. While P3 to P5 (16.67%, 25%, and 33.33%) of PPXL content improved slightly, it disintegrated after more than 30 min.

The CCS level determines the DT. The lower CCS content Formulations C1 to C3 (5%, 10%, and 15%) performed slightly better and disintegrated after 30 min. While a higher CCS content of 25%, C4) resulted in a significant reduction, the pellets exploded and disintegrated into smaller pieces within 2 min. This was possibly due to the swelling effect of CCS disintegration, which forced the pellets to explode and facilitate water entry. ^18^ However, the formula failed the shape test because of pellet shape distortion. Consequently, the shape of pellets must be improved.

The binder and disintegrant often influence DT. The binder was used at a fixed value (0.5%). The DT fluctuated based on the disintegrant concentrations in the formulation, with an inverse relationship with the disintegrant. PPXL has good hydration ability and high capillary efficiency by wicking, with little swelling effect compared to CCS. ^4^

The super-disintegrant CCS affected friability and DT. CCS swelling was observed during granulation of the dry blend, which was directly related to the amount of CCS employed in the formulation. Regardless of the binder concentration, the higher the proportion of CCS employed, the more swelling occurred, resulting in the formation of more fines during spheronization. In addition, CCS expands as it comes into contact with water, necessitating more water for pelletization. ^4^ In a trial to enhance the P1 formulation, which had better shape roundness > 0.939, because PP XL alone was insufficient to meet DT’s needs, PE1 ( Table 1) was prepared using 99% ethanol 50/50 v/v of water to granulate the dry mixture, but the wet mass resembled chewing gum and did not extrude, and no pellets were produced.

### 3.5 Development of pellets with combination of disintegrants (CP1-CP12)

Another attempt was made to obtain fast-disintegrating pellets with a desirable shape by taking advantage of the potential synergistic behavior of disintegrants with diverse principles of action, such as swelling and water wicking. ^27^

Several pellet formulations were prepared using a combination of CCS and PPXL, as described in CP1 to CP12 ( Table 1). Based on these results, the combination of PPXL and CCS negatively affected sphericity. The CP1 to CP11 formulations ( Table 2) showed no shape enhancement, as evidenced by a pellet image with a clear roughness and distortion of the surface and a high percentage of fine particles. Only CP12 achieved the required spherical and smooth surface pellets.

The results of pellet evaluation for the DT of different formulations showed that while the CP1 formulation improved slightly, it disintegrated after more than 30 min ( Table 2).

The Second formulation, CP2, CP3, and CP4, was prepared by increasing the CCS concentration with a fixed concentration of PPXL. CP2 and CP3 improved slightly but disintegrated after more than 30 min. While CP4 achieved a desirable DT (less than 2 min), it was noticeable that using 15% of CCS positively affected DT.

Another formulation, CP5, was prepared by increasing PPXL with fixed CCS content. Again, no significant change in DT was observed.

Other formulations, CP6, CP7, and CP8, were prepared by increasing the MCC content and lowering the mannitol content while keeping PPXL and CCS concentrations constant in CP2, CP3, and CP5, respectively. Again, there were no notable changes in the DT.

In another formulation, CP9 and CP10 were made with an equal amount (15%) of PPXL and CCS, and faster DT was observed (less than 30 min.)

The last formulations, CP11 and CP12, were prepared by increasing PEG content to (20%) and using a fixed percentage of CCS (15%), 15%, and 5% PPXL, respectively. and The results showed a clear improvement in DT (less than 2 min.). In addition, we noticed in CP9–CP11 that the extrudes were fragile; therefore, a low-speed spheronizer of 1000 RPM was used.

We also observed that increasing the PEG 400 concentration to 20% w/w resulted in smaller, more spherical, smoother pellets. This result is supported by a study that indicated that combining hydrophilic polymers with Avicel’s lower wet mass consistency allows for easier extrusion, resulting in spherical, smoother pellet surfaces and smaller pellet sizes. ^5^

The pellet composition significantly affected the DT of all formulations. Both the hydrophilicity of PEG and the solubility of mannitol had a limited ability to disintegrate the matrix of the pellets, but when combined with CCS and PPXL, they increased pellet disintegration by swelling and wetting of the pellet core. The combination of these approaches has a synergistic effect on pellet formation, thereby overcoming the problem of drug disintegration in extruded MCC pellets. ^27^

CP12 was considered a successful formula, and it was used to load model drugs. The particle size distribution results for CP12 are listed in ( Table 3). A sieve shaker was used to perform these tests. The results revealed that the majority (82.67%) of the CP12 batch pellets ranged from 600 to 850 μm. ^28^ Consequently, this size fraction was selected for further studies. Pellets usually come in a range of (0.5-2) mm. ^6^ This indicates that the outcomes were satisfactory.

**Table 3.** Results of placebo CP12 pellets size distribution by sieve analysis.

Mesh size number	Sieve #	Weight retained on each sieve (g)	Percent retained on each sieve (%)	Cumulative percent retained on each sieve (%)	Percentage passing (%)
16	6	0.03	0.26	0.26	99.74
20	5	0.04	0.34	0.60	99.40
30	4	9.59	82.67	83.28	16.72
40	3	1.21	10.43	93.71	6.29
60	2	0.49	4.22	97.93	2.07
Pan	1	0.24	2.07	100.00	00

% retained = (Weight of pellets retained over x# sieve/Actual weight of pellets) *100. Pellets weight = 11.6 gm.

**Figure 1.**
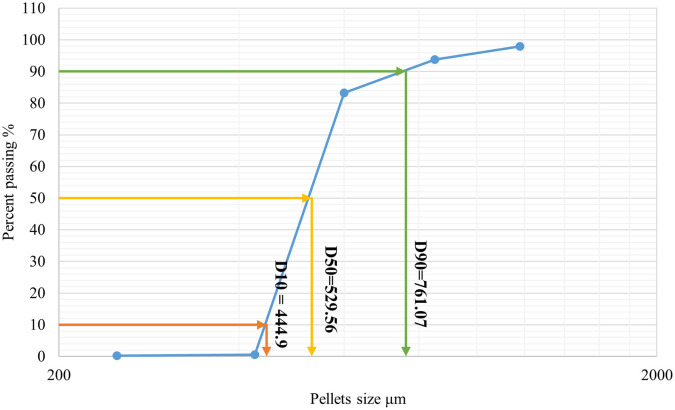
Formula CP12 particle size distribution by sieving.

As shown in ( Figure 1) 10% of the samples were smaller than 444.9 μm, 50% were smaller than 529.56 μm, and 90% were smaller than 761.07 μm. The size and shape analysis results are presented in ( Table 4). The test was performed using image j ^®^ free software. The pellets in most CP12 batches were approximately spherical with a roundness range between (0.88-0.93).

**Table 4.** Results of placebo CP12 size and shape analysis.

Pellet group #	Average Area = $A=\pi r^{2}$ (μm2)	A/π	R = SQRT (A/π)	D (μm) = R*2	P.	F. D. (μm)	C.	AR	RN.	Microscopic image
1	510857	162611	403	807	2687	871	0.89	1.10	0.91	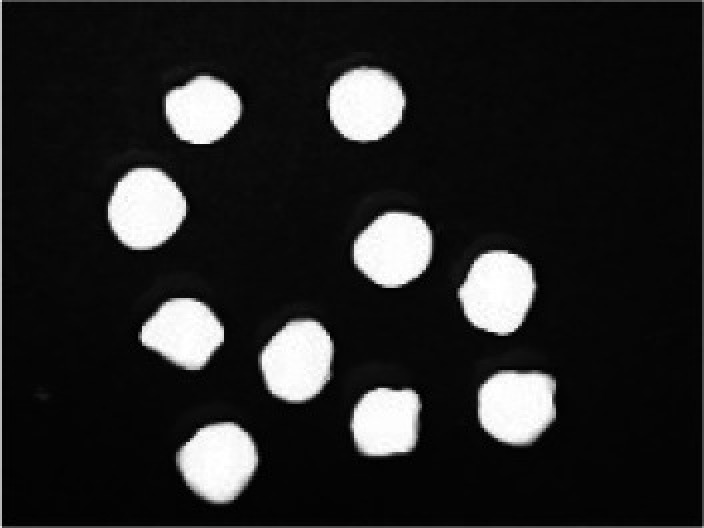
2	394223	125485	354	708	2362	771	0.89	1.12	0.90	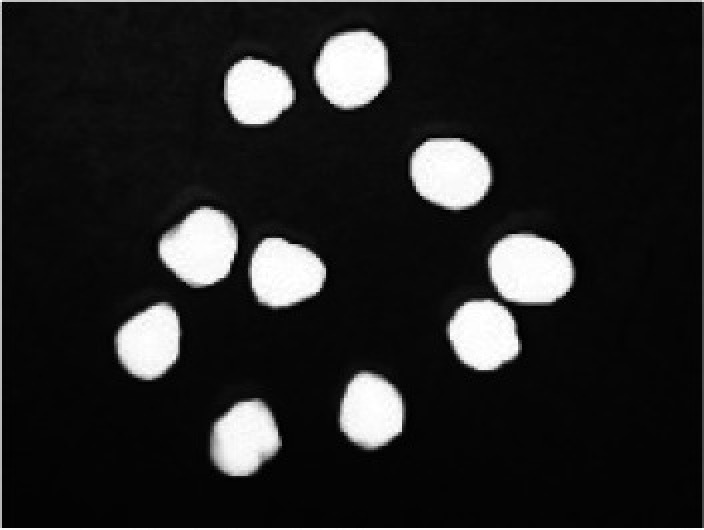
3	434759	138388	372	744	2498	816	0.87	1.14	0.88	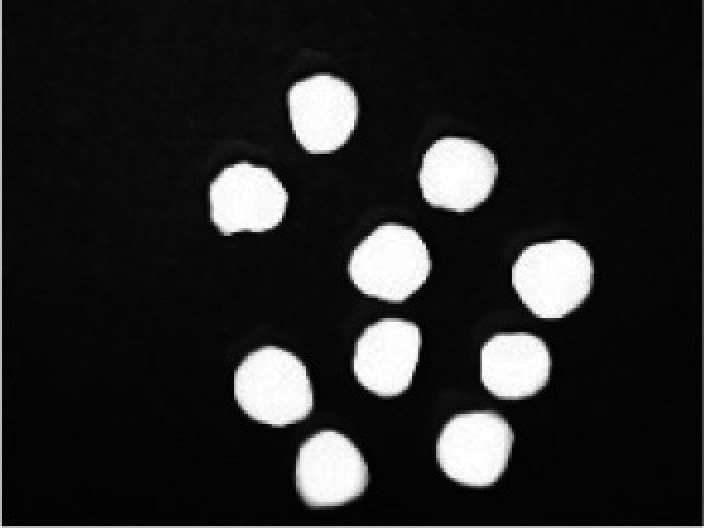
4	480388	152912	391	782	2669	855	0.85	1.13	0.89	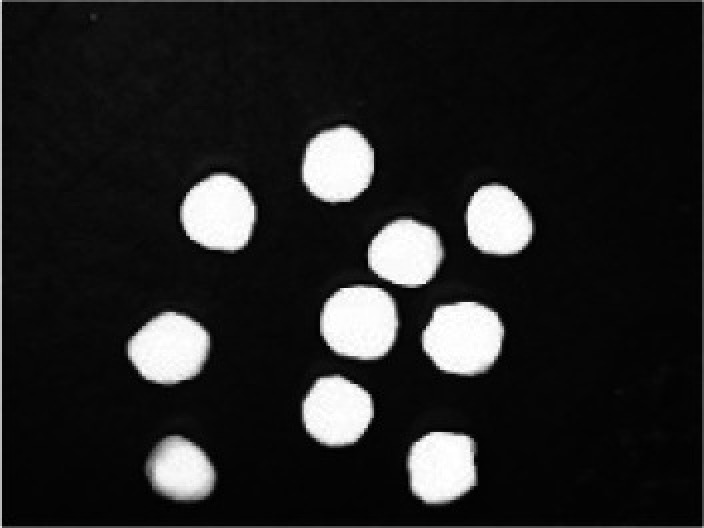
5	454227	144585	380	760	2542	830	0.88	1.11	0.90	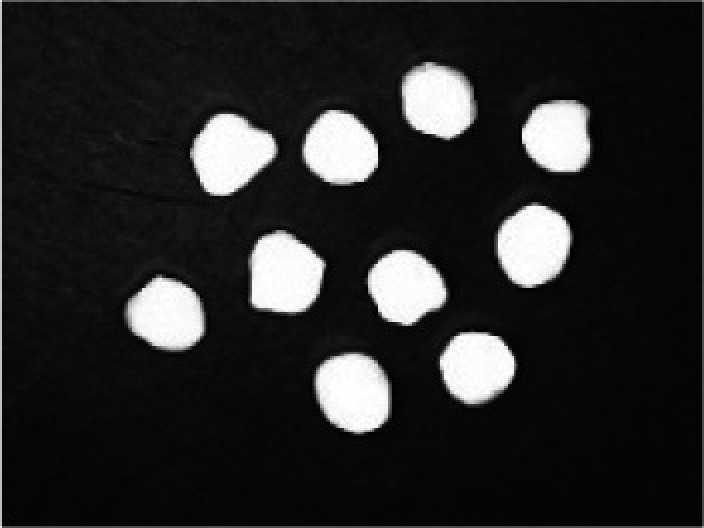
6	459254	146185	382	765	2547	819	0.89	1.08	0.93	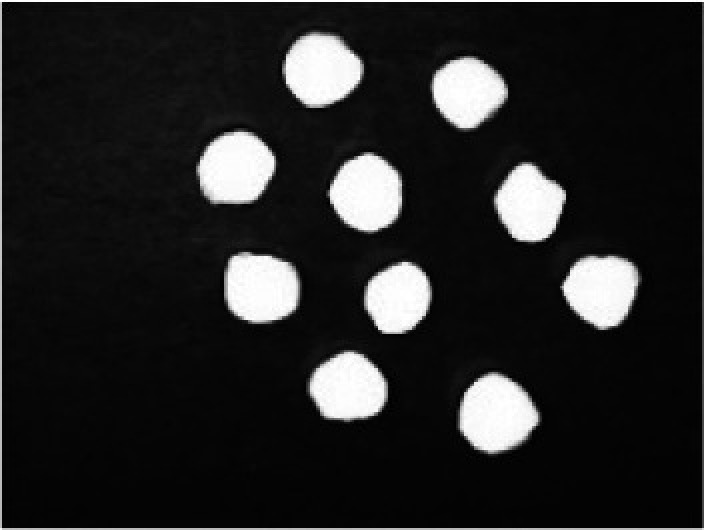
7	486055	154716	393	787	2620	852	0.89	1.12	0.90	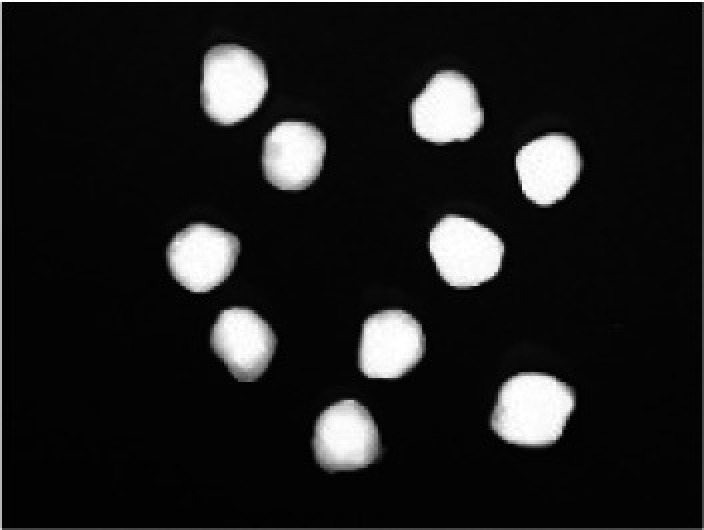
8	496802	158137	398	795	2669	868	0.87	1.09	0.92	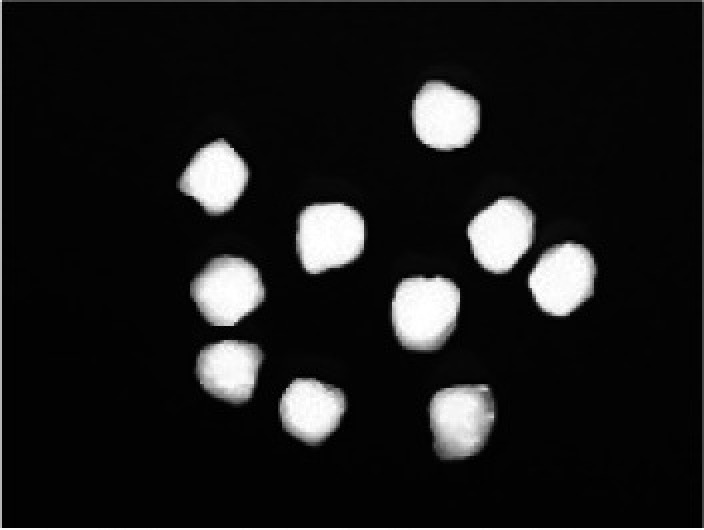
9	443173	141066	376	751	2527	819	0.87	1.11	0.90	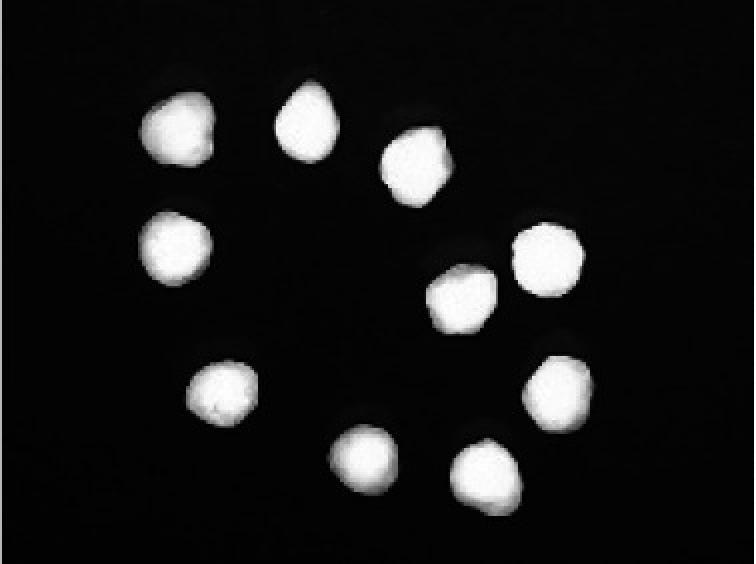
10	476108	151550	389	779	2620	846	0.87	1.10	0.91	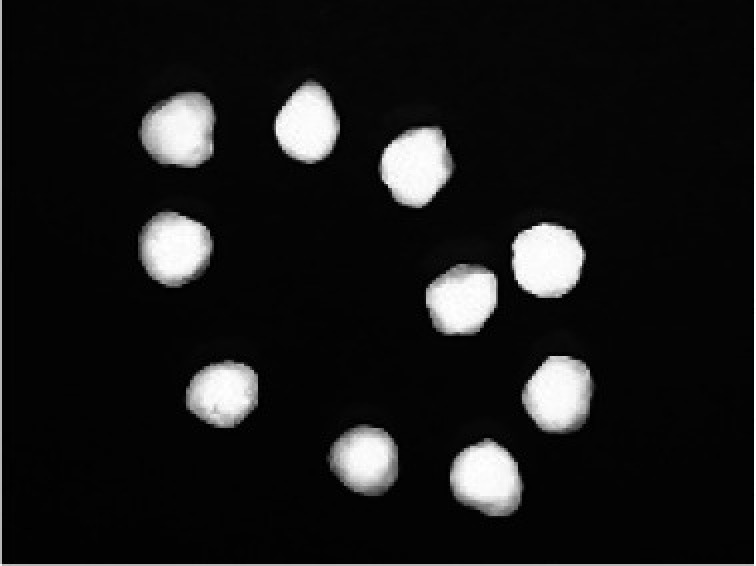

A: Area, SQRT: Square root, D: Diameter, R: Radius, F. D.: Ferret diameter, P: Perimeter, C: Circularity, AR: Aspect ratio, RN.: Roundness.

All CP12 pellets have an aspect ratio in the range of (1.08–1.14), which is within the limit (an aspect ratio of 1.00 denotes an ideal spherical shape; in practice, values up to 1.2 are allowed). ^18^

### 3.6 Development of pellets with active ingredient

CP12 was considered a successful formula and was used to load the drugs at various percentages. Pseudoephedrine hydrochloride was used as a model drug, which is freely soluble in water (CPP1- CPP4, Table 1). Orphenadrine citrate, sparingly soluble in water (CPO1 **–** CPO4, Table 1), was used as a second model drug. The DT of several batches is shown, as the formulation offers a desirable shape and fast DT. These formulations were also assessed using assays and dissolution studies for each API. Numerous experiments have been performed to gradually increase the percentage of drugs. Finally, pellets containing 30% of the drug were effectively prepared (CPP4, CPO4).

**Figure 2.**
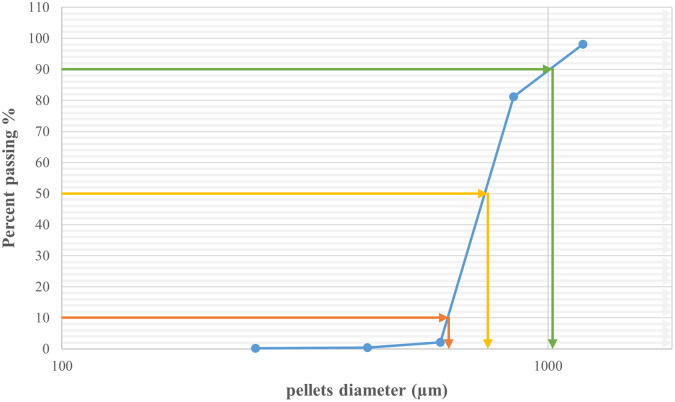
Pseudoephedrine hydrochloride pellets size distribution by sieve analysis CPP4.

The particle size distribution results for CPP4 are listed in ( Table 5). The results revealed that the majority (79.14%) of the CPP4 batch pellets ranged from 600 to 850 μm. Consequently, this size fraction was selected for further study. As shown in ( Figure 2), 10% of the samples were smaller than 625.08 μm, 50% were smaller than 751.44 μm, and 90% were smaller than 1021.53 μm.

**Table 5.** Results of pseudoephedrine hydrochloride pellets size distribution by sieve analysis CPP4.

Mesh size number	Sieve #	mesh size (μm)	weight retained on each sieve (g)	Percent retained on each sieve (%)	Cumulative percent retained on each sieve (%)	Percentage passing (%)
16	6	1180	0.2	1.87	1.87	98.13
20	5	850	1.81	16.93	18.8	81.2
30	4	600	8.46	79.14	97.94	2.06
40	3	425	0.18	1.68	99.62	0.38
60	2	250	0.02	0.19	99.81	0.19
Pan	1	------	0.02	0.19	100.00	00

Pellets weight = 10.69 gm.

The results of the size and shape analysis of the CPP4 batch are shown in ( Table 6). The test was performed by image J ^®^ free software. The pellets in the majority of the CPP4 batch were approximately spherical with a roundness range of between (0.85-0.91).

**Table 6.** Results of pseudoephedrine hydrochloride size and shape analysis CPP4.

Pellet group #	Average Area = R2 π (μm2)	A/π	R = SQRT (A/π)	D (μm) = R*2	P.	F.D. (μm)	C.	AR	RN.	Microscopic image
1	511323	162759	403	807	2742	905	0.85	1.17	0.86	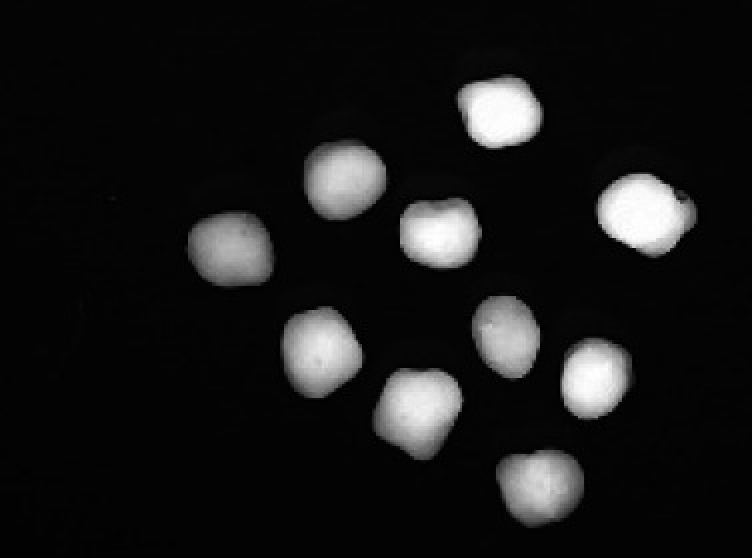
2	407117	129589	360	720	2421	808	0.87	1.16	0.87	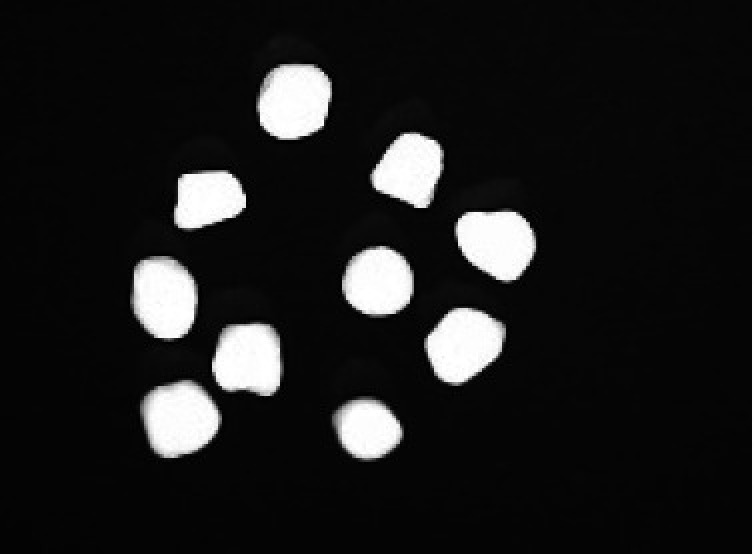
3	407197	129615	360	720	2427	809	0.87	1.17	0.86	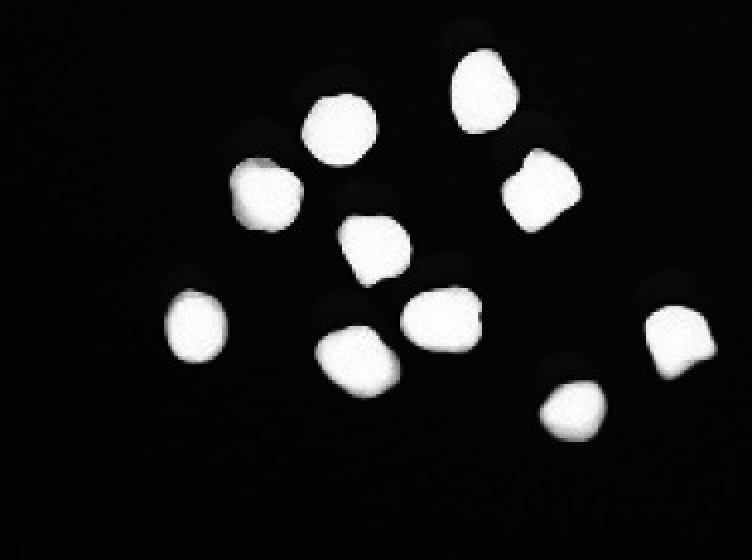
4	450253	143320	379	757	450253	837	0.88	1.17	0.86	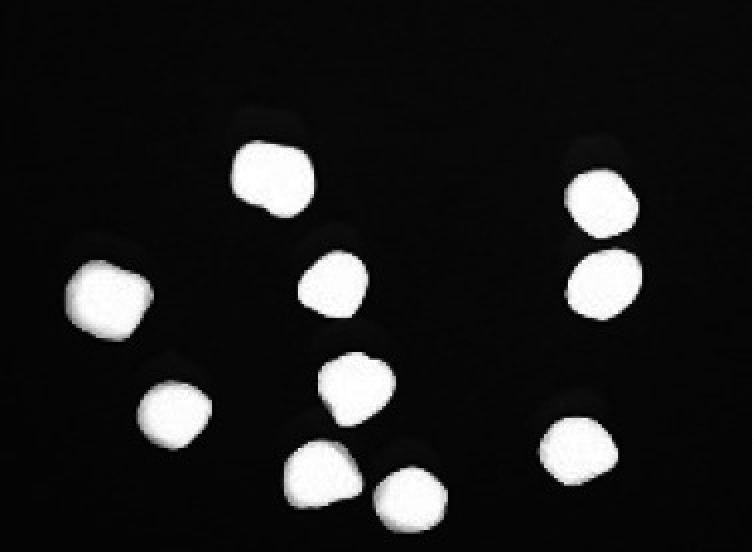
5	447933	142581	378	755	2528	836	0.88	1.16	0.87	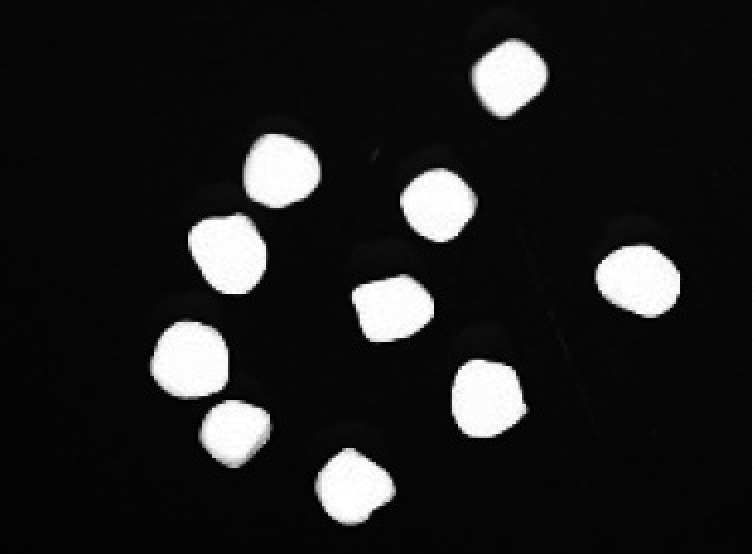
6	435306	138562	372	744	2492	818	0.88	1.12	0.90	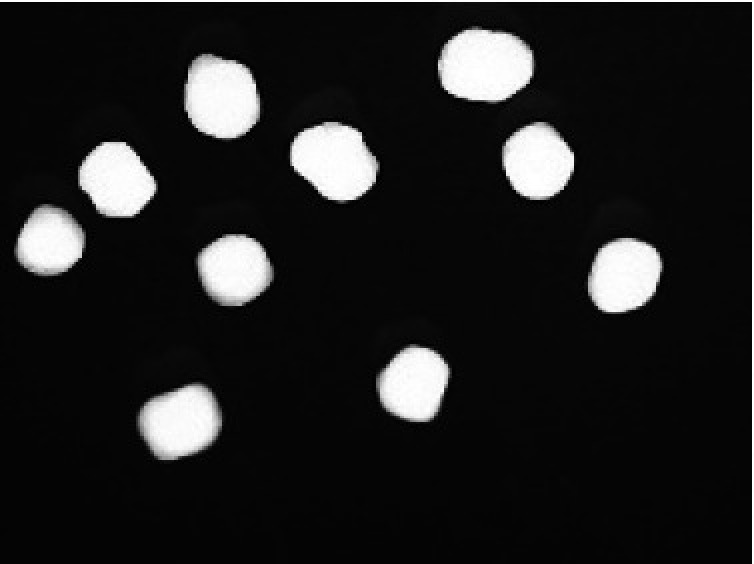
7	427212	135986	369	738	2477	820	0.87	1.18	0.85	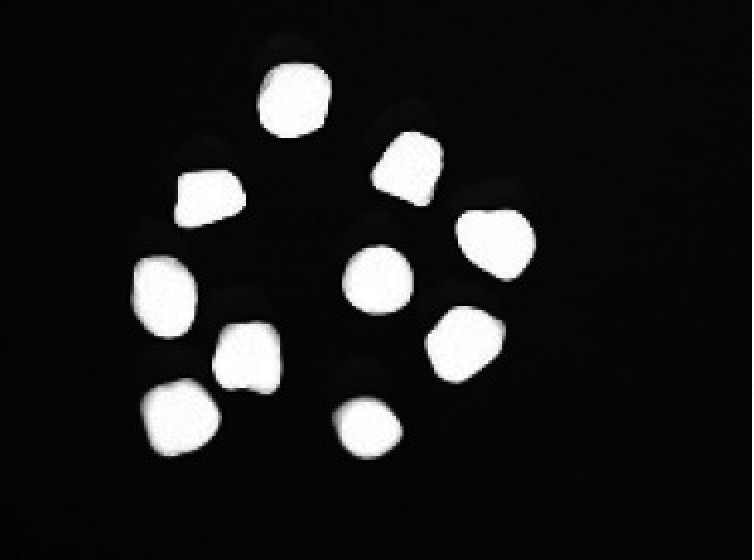
8	410837	130774	362	723	2403	786	0.89	1.10	0.91	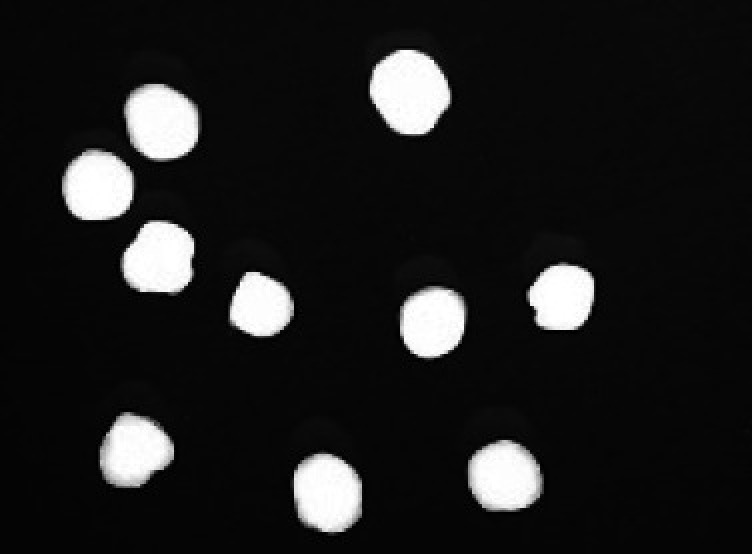
9	446720	142195	377	754	2535	827	0.87	1.12	0.90	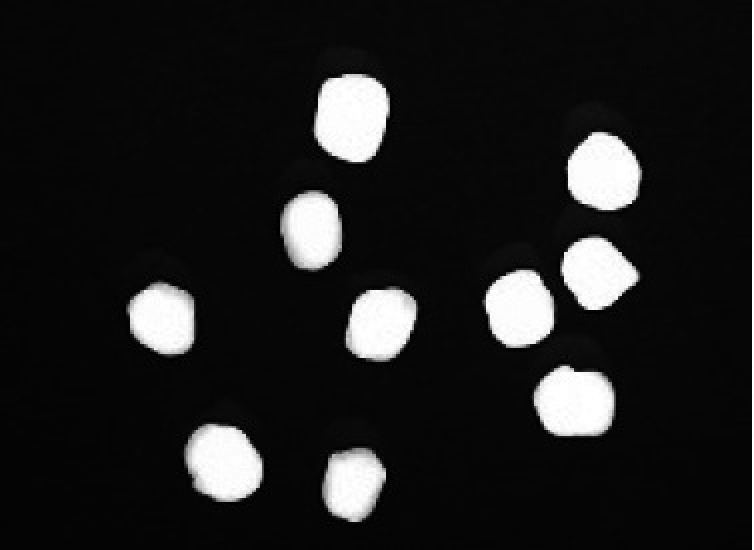
10	419091	133401	365	730	2442	810	0.88	1.16	0.87	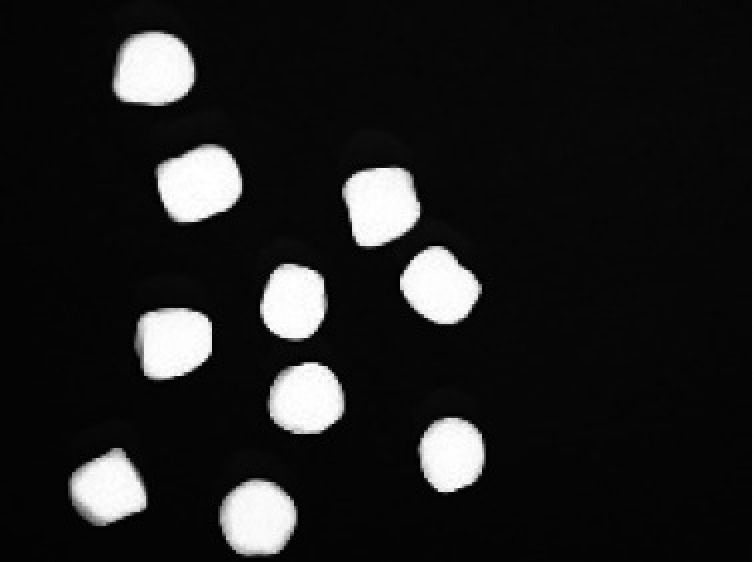

All pellet formulations have an aspect ratio of (1.10–1.18), which is within the limit (an aspect ratio of 1.00 denotes an ideal spherical shape; in practice, values up to 1.2 are allowed). ^18^

**Figure 3.**
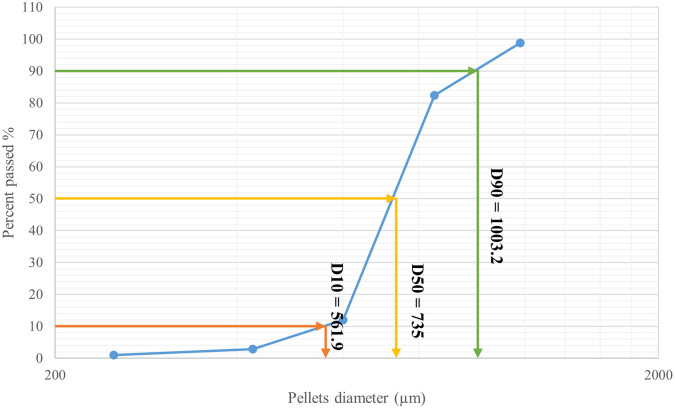
Orphenadrine citrate pellets size distribution by sieve analysis CPO4.

The particle size distribution results for CPO4 are listed in ( Table 7). The results revealed that the majority (70.35%) of the CPO4 batch pellets ranged from 600 to 850 μm. Consequently, this size fraction was selected for further studies. As shown in ( Figure 3) 10% of the samples were smaller than 561.9 μm, 50% were smaller than 735 μm, and 90% were smaller than 1003.2 μm.

**Table 7.** Results of orphenadrine citrate pellets size distribution by sieve analysis CPO4.

Mesh size number	Sieve #	mesh size (μm)	weight retained on each sieve (g)	Percent retained on each sieve (%)	Cumulative percent retained on each sieve (%)	Percentage passing (%)
16	6	1180	0.05	1.18	1.18	98.82
20	5	850	0.7	16.47	17.65	82.35
30	4	600	2.99	70.35	88.00	12.00
40	3	425	0.39	9.18	97.18	2.82
60	2	250	0.08	1.88	99.06	0.94
Pan	1	------	0.04	0.94	100	00

Pellets weight = 4.25 gm.

The results of the size and shape analysis of the CPO4 batch are shown in ( Table 8). The test was performed by image j free software. The pellets in the majority of the CPO4 batch were approximately spherical with a roundness range between (0.87-0.91).

**Table 8.** Results of orphenadrine citrate size and shape analysis CPO4.

Pellet group #	Average Area = R2 π (μm2)	A/π	R = SQRT (A/π)	D (μm) = R*2	P.	F.D. (μm)	C.	AR	RN.	Microscopic image
1	523524	166643	408	816	2730	897	0.88	1.12	0.90	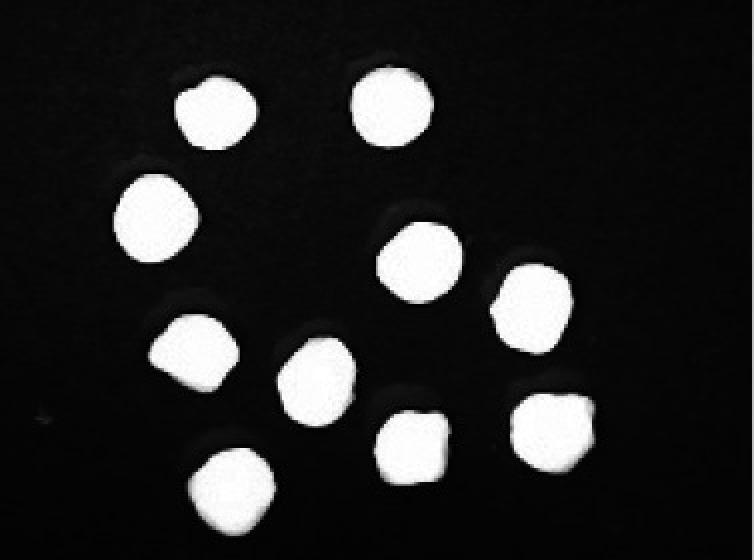
2	482842	153693	392	784	2622	869	0.88	1.15	0.87	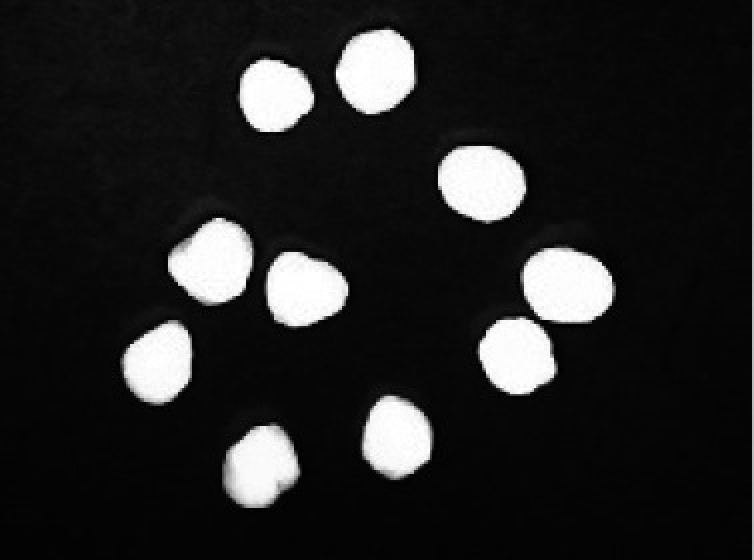
3	523097	166507	408	816	2723	879	0.89	1.10	0.91	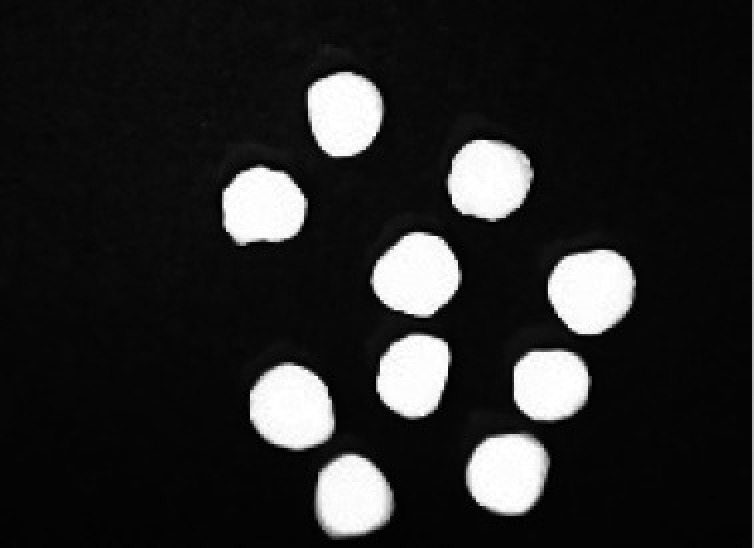
4	484375	154181	393	785	2620	850	0.88	1.10	0.91	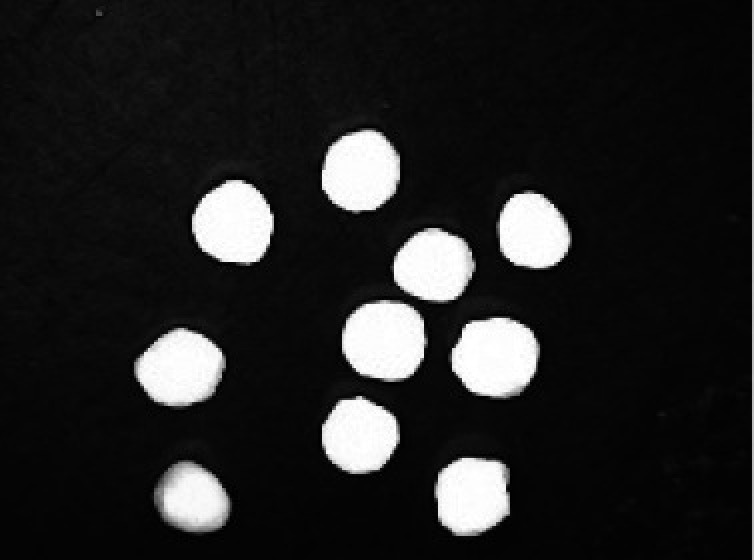
5	522031	166168	408	815	2734	892	0.88	1.11	0.90	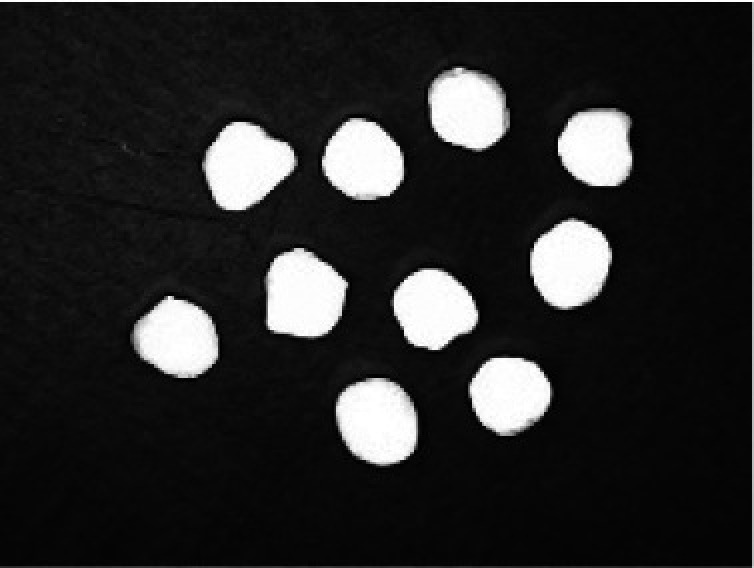
6	491335	156397	395	791	2658	863	0.87	1.09	0.92	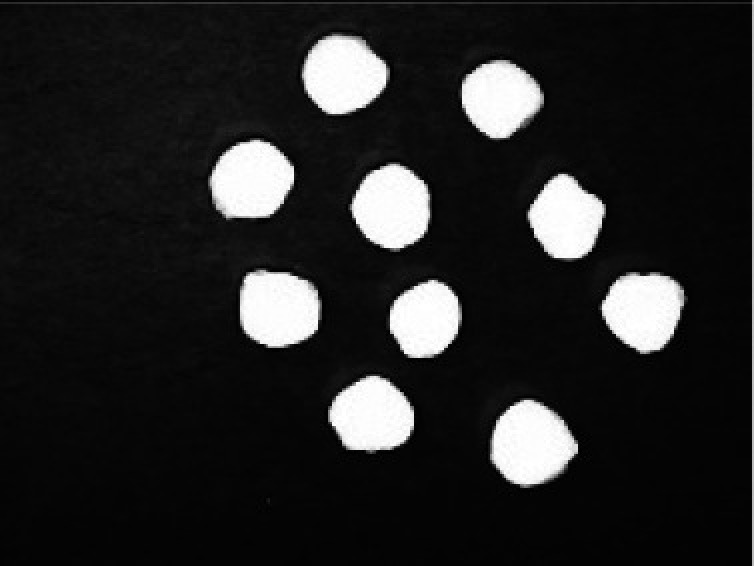
7	458107	145820	382	764	2558	848	0.88	1.14	0.88	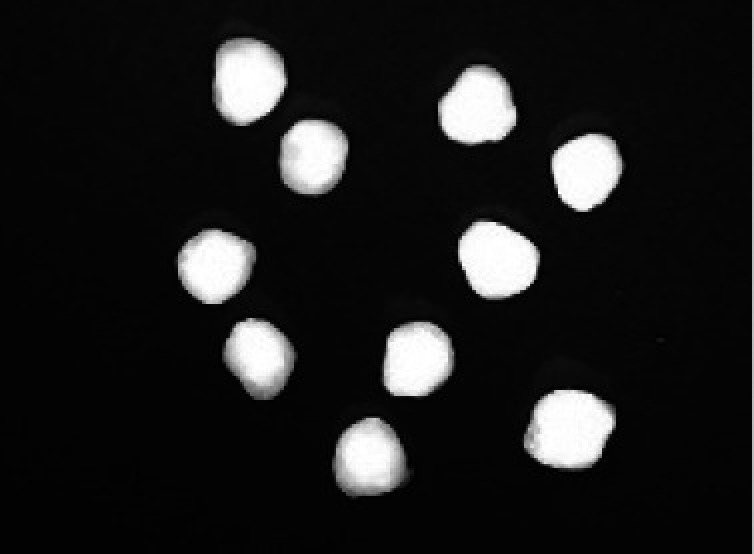
8	436906	139071	373	746	2514	836	0.87	1.12	0.90	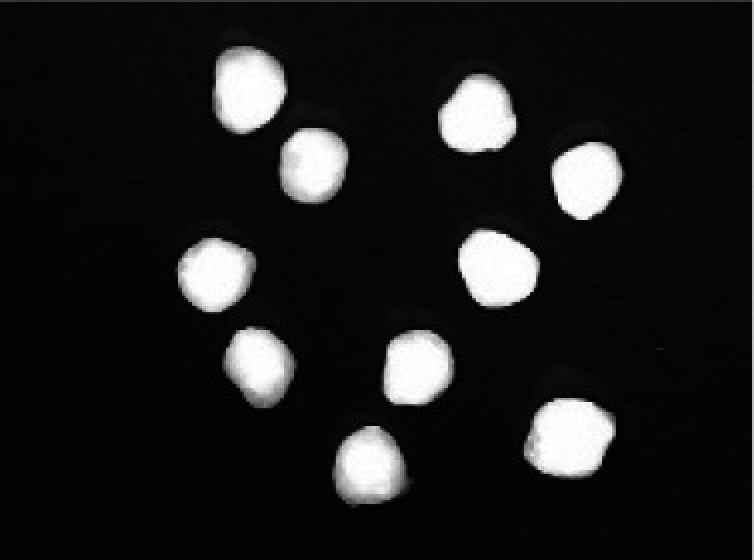
9	410304	130604	361	723	2430	798	0.87	1.13	0.89	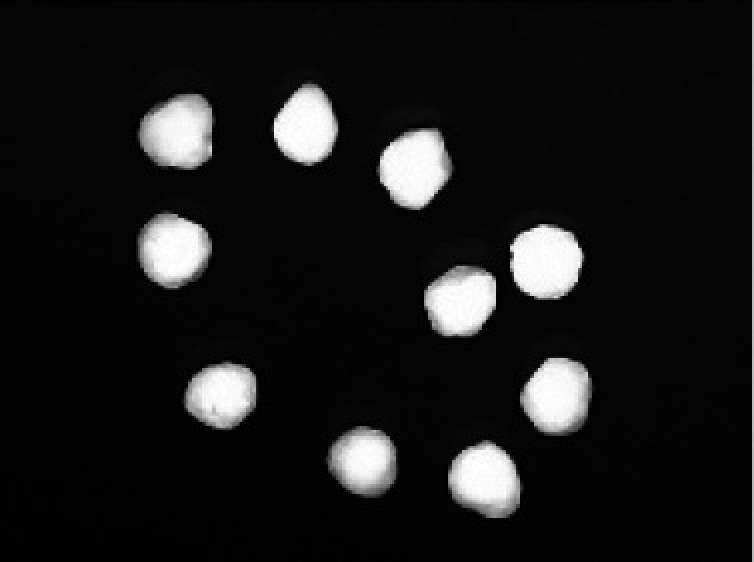
10	410304	130604	361	723	2430	798	0.87	1.13	0.89	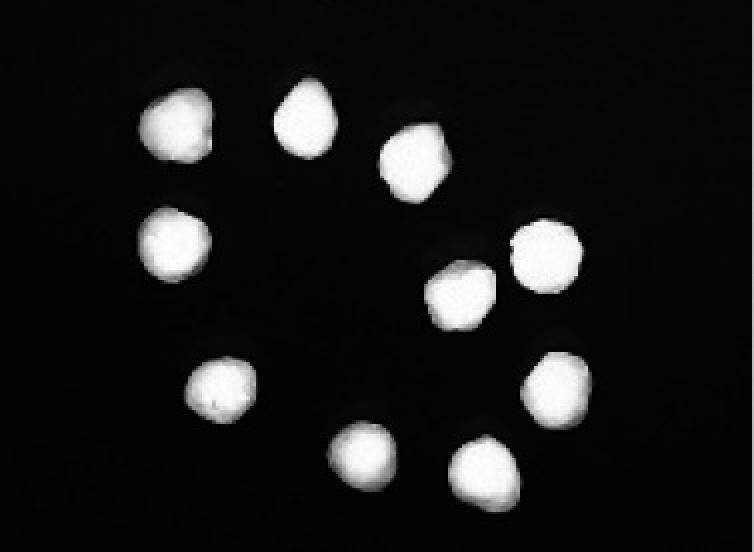

All pellet formulations have an aspect ratio of 1.09–1.15, which is within the limit (an aspect ratio of 1.00 denotes an ideal spherical shape; in practice, values up to 1.2 are allowed). ^18^

**Figure 4.**
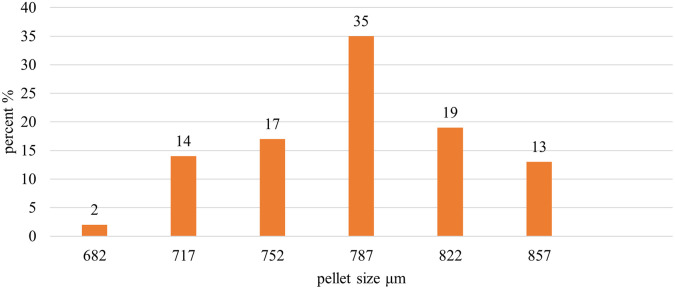
Placebo CP12 pellets size distribution by image j® software.

**Figure 5.**
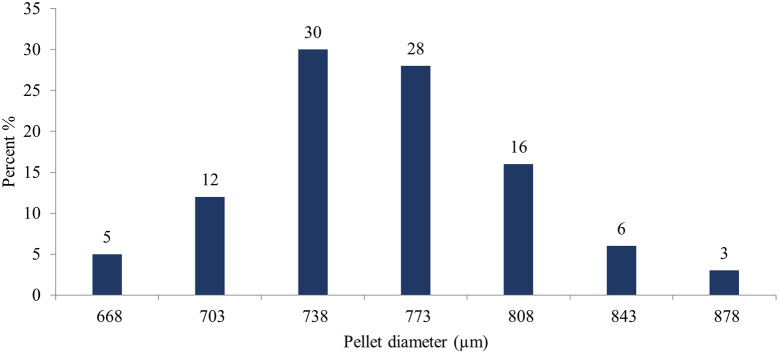
Pseudoephedrine hydrochloride pellets size distribution by image j® software CPP4.

**Figure 6.**
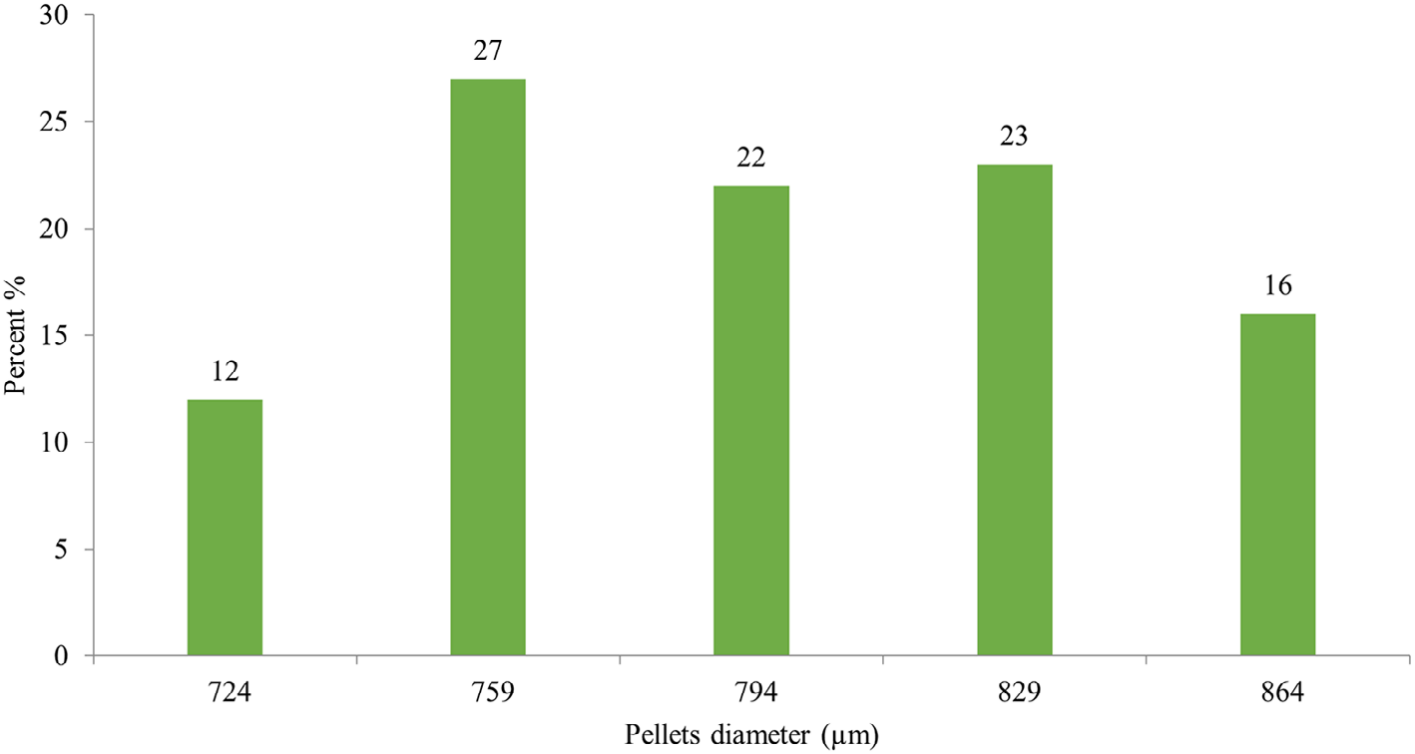
Orphenadrine citrate pellets size distribution by image j® software CPO4.

Image J ^®^ software analyzed the pellet size distribution for the formulas CP12, CPP4, and CPO4. The pellet size distributions for formulas CPP4 and CPO4 are shown in Figures 4, 5, and 6, respectively. For the formula CP12, 85% of the samples had a diameter range of (717-822 μm) indicating that the sample has a narrow size distribution. For formula CPP4, 85% of the samples had a diameter range (703–808 μm), indicating that the sample had a narrow size distribution. For formula CPO4, 84% of the sample had a diameter range (724–829 μm), indicating the sample has a narrow size distribution.

***3.6.1 Pellets yield***

**Figure 7.**
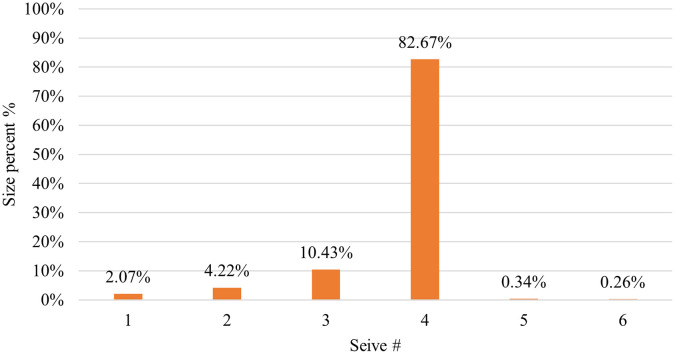
Results of CP12 pellets yield.

The pellet yield of CP12 by sieve analysis for 600-850 μm is depicted in ( Figure 7) and was excellent (82.67%).

**Figure 8.**
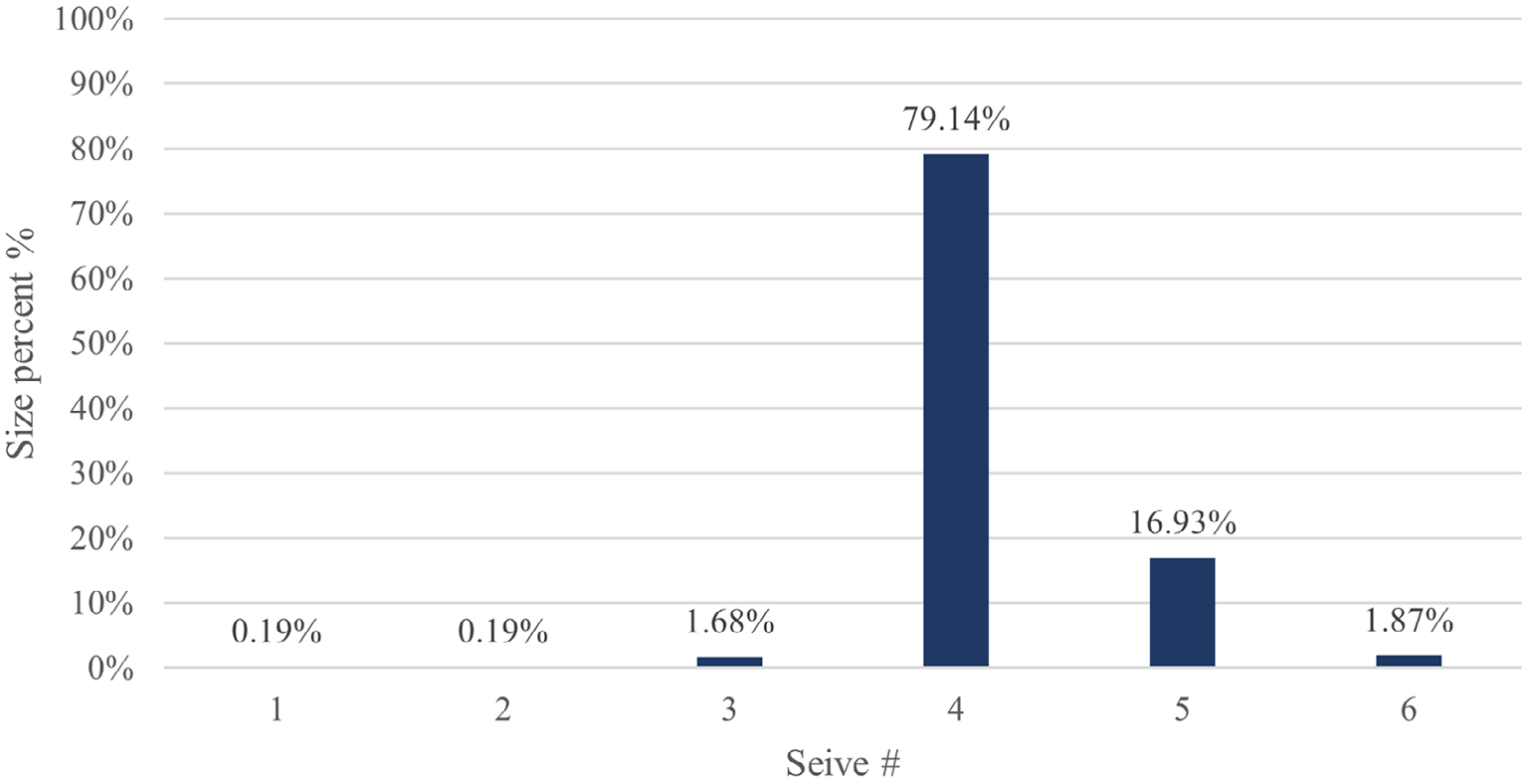
Results of pseudoephedrine hydrochloride pellets yield CPP4.

The yield of the CPP4 by sieve analysis for 600-850 μm is depicted in ( Figure 8), which is terrific (79.14%).

**Figure 9.**
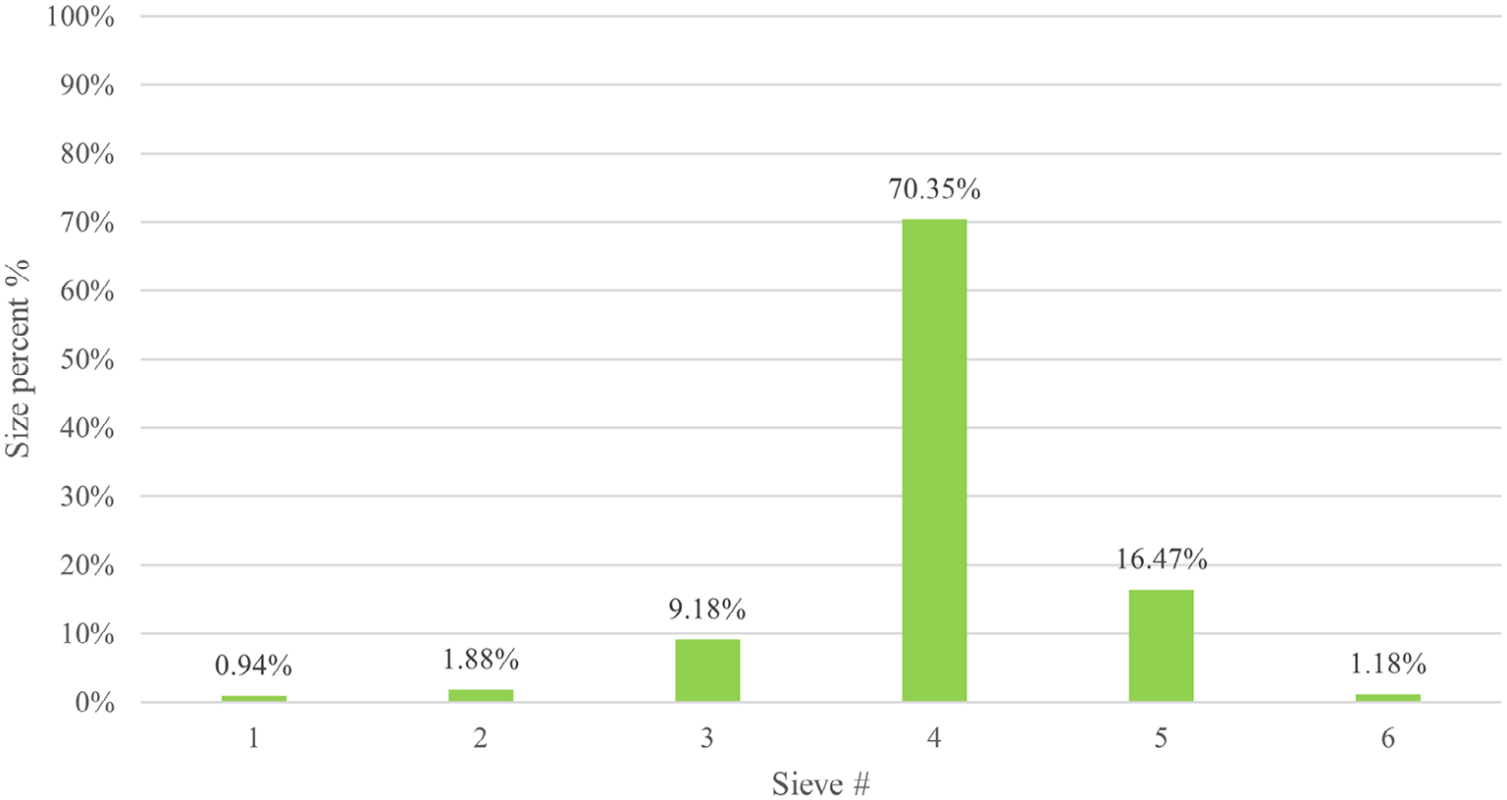
Results of orphenadrine citrate pellets yield CPO4.

Pellet yield of CPO4 by sieve analysis for 600-850 μm is shown in ( Figure 9) and is satisfactory (70.35%). ^28^

***3.6.2 Moisture content***

The scale directly reports the percentage of weight loss due to moisture loss. Although CP12 placebo pellets L.O.D = 4.6, Pseudoephedrine hydrochloride L.O.D = 5.83, and Orphenadrine citrate L.O.D = 5.36 are acceptable, high moisture content deteriorates disintegration.

***3.6.3 Friability***

The friabilities of CP12, CPP4, and CPO4 were estimated to be (0.6%, 0.65%, and 0.71%) within the acceptable limits (less than 1%).

***3.6.4 Camera capture of the pellet disintegration process***

Disintegration was evaluated at room temperature under static conditions. The camera captured images every 30 s ( Table 9), illustrating that MCC pellet X3 with mannitol and PEG 400 did not disintegrate. Within 120 s, cracks appeared in P5 pellets containing mannitol, PEG, and PPXL. As seen in the C4 pellets, they begin to explode into many fragments within 30 s. Moreover, the CP12 pellets containing PEG 400, mannitol, CCS, and PPXL began to explode into many loosely linked particles after 60 s, which quickly separated under the oscillating motion of the USP disintegration equipment. The photographs are compatible with the results mentioned above for the USP disintegration device. When the temperature was increased to 37°C, the disintegration caused the split into tiny fragments.

**Table 9.** Camera capture of pellet disintegration at different time intervals.

Pellet #	0 sec.	30 sec.	60 sec.	90 sec.	120 sec.
X3	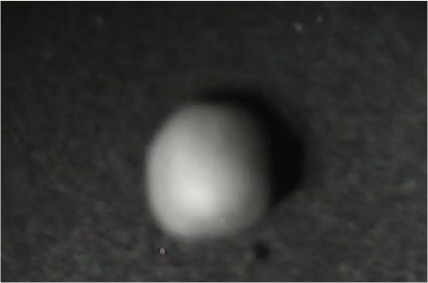	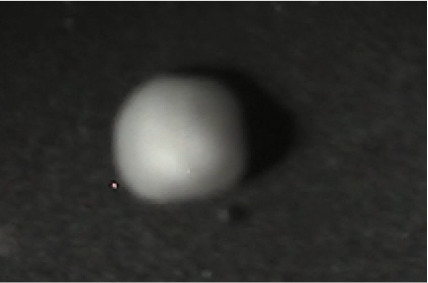	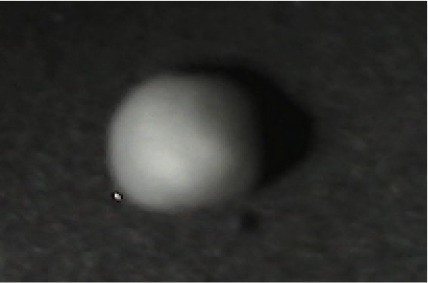	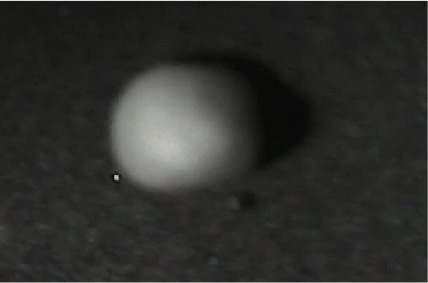	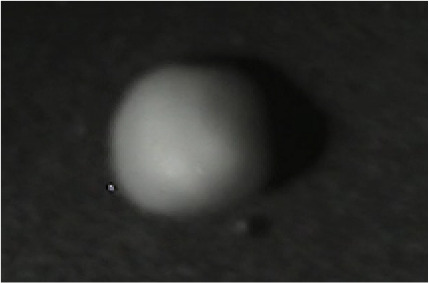
P5	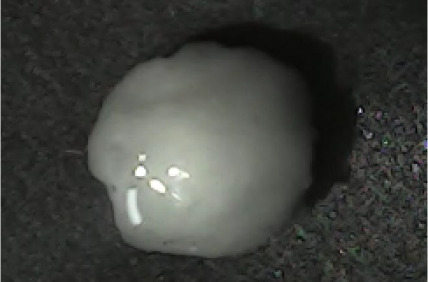	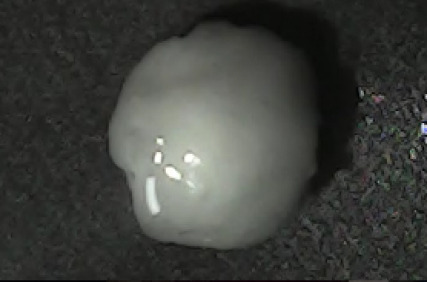	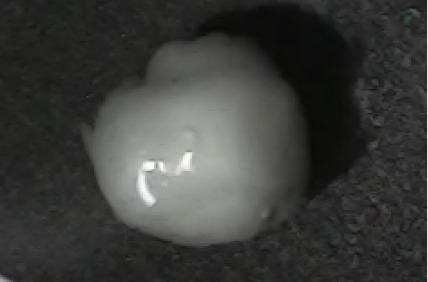	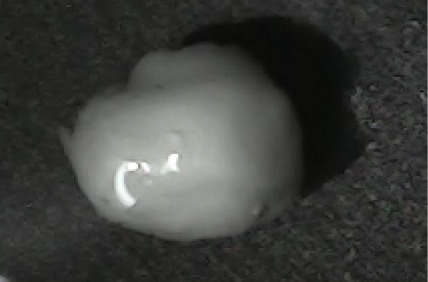	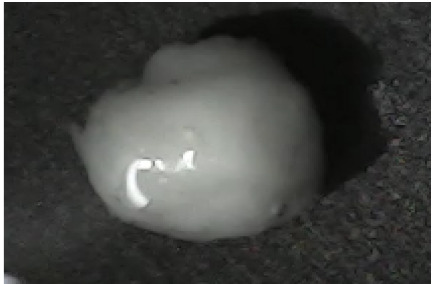
C4	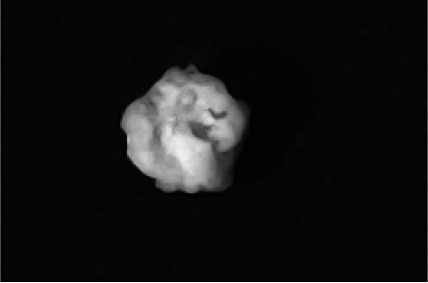	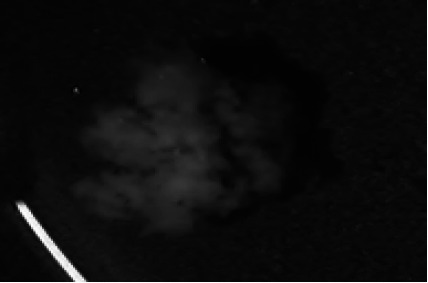	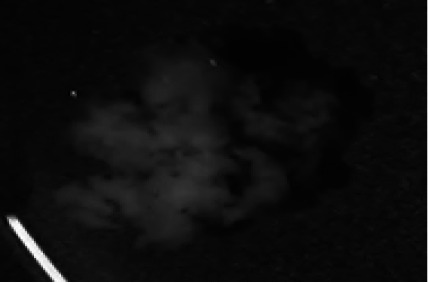	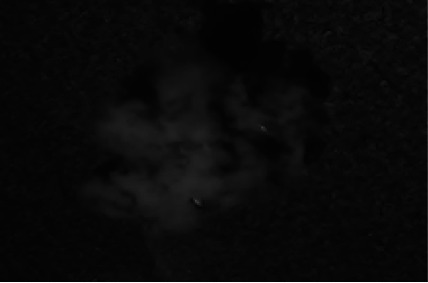	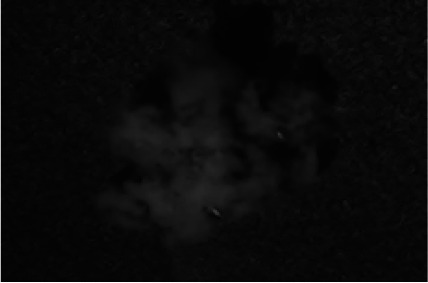
CP12	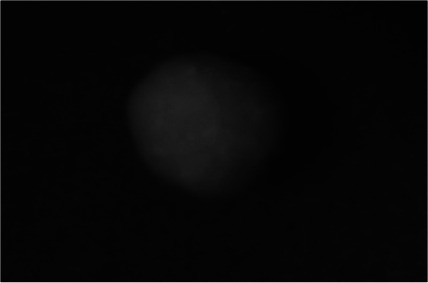	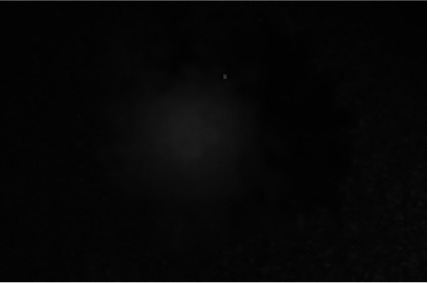	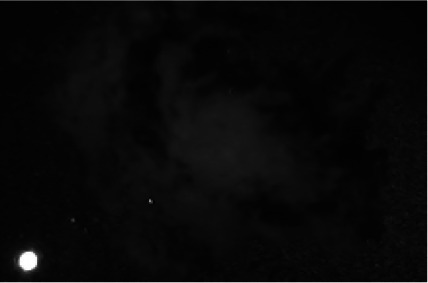	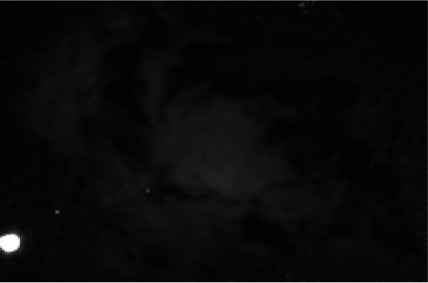	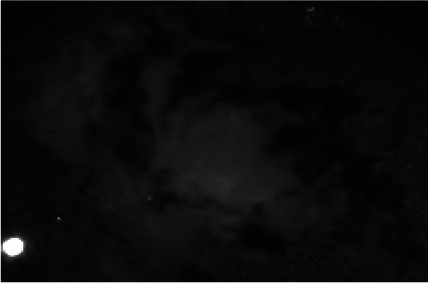
CPP4	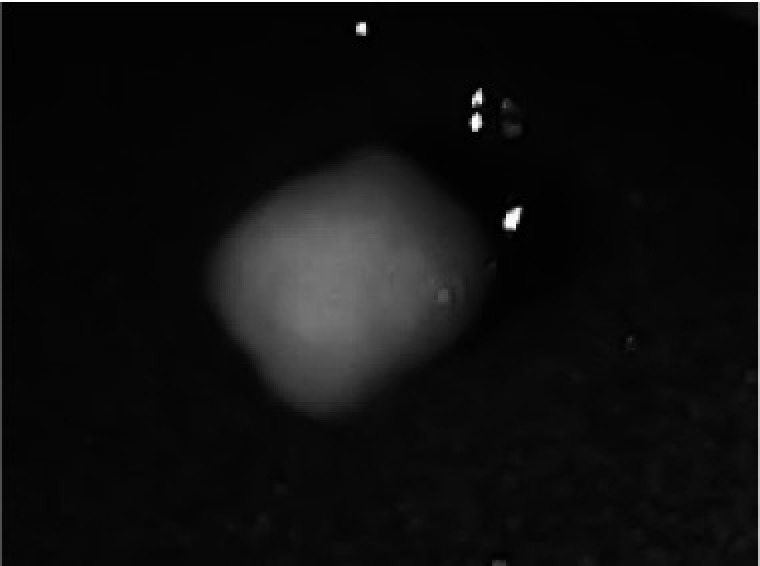	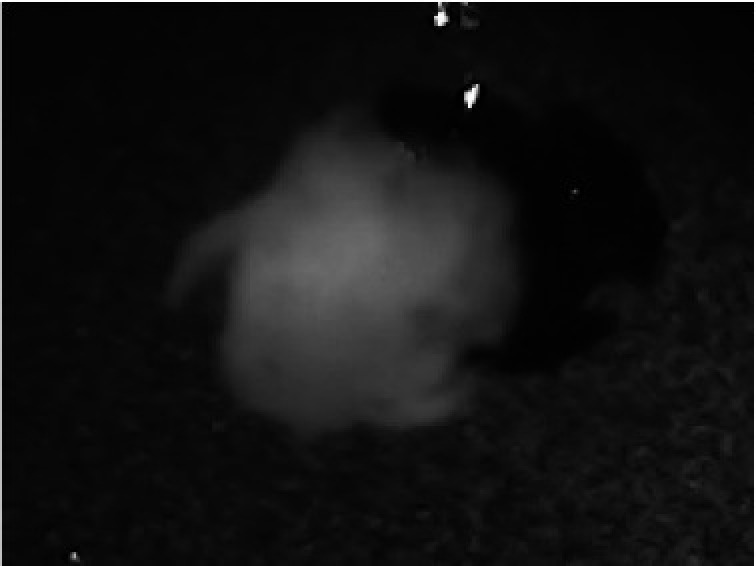	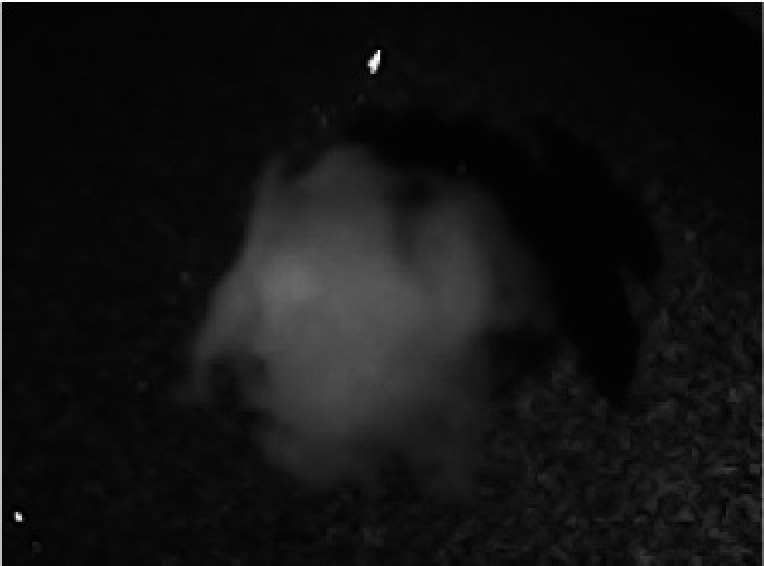	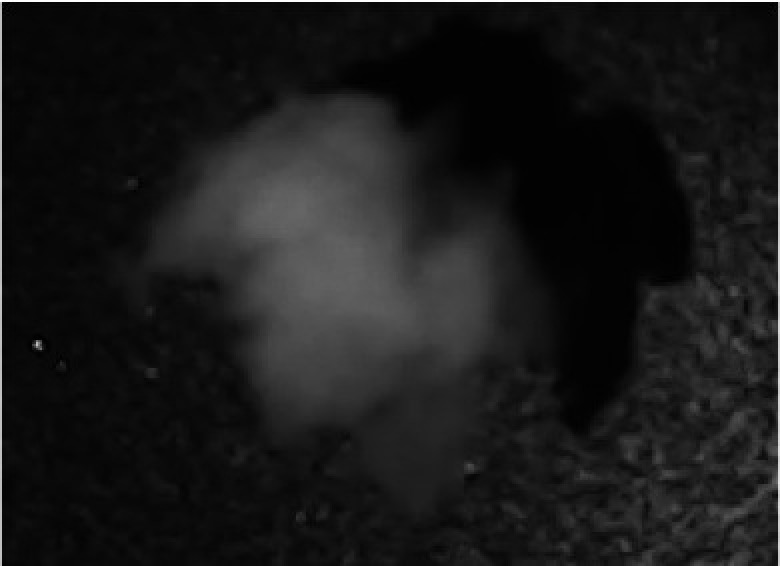	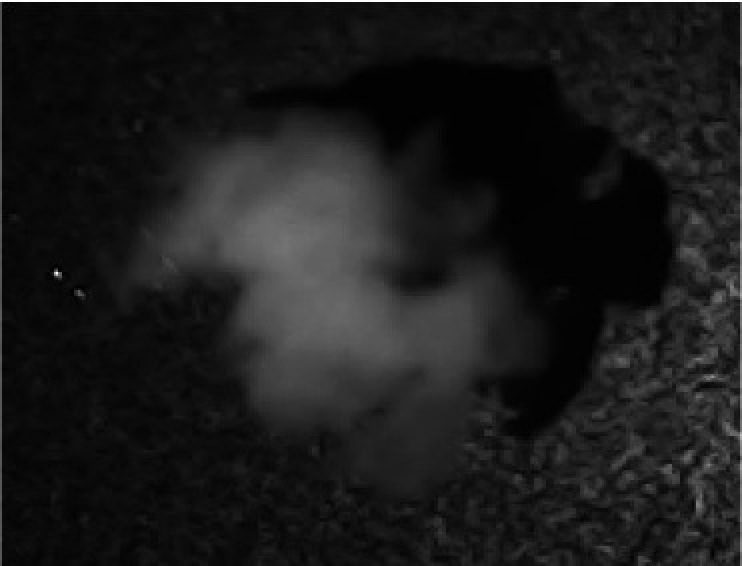
CPO4	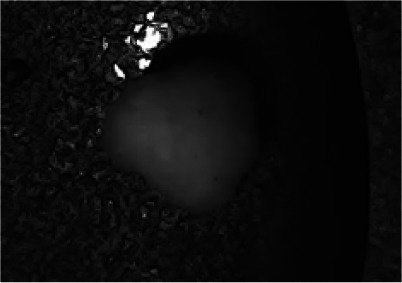	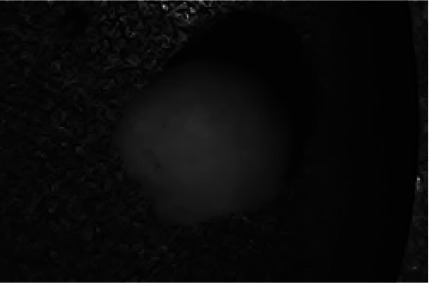	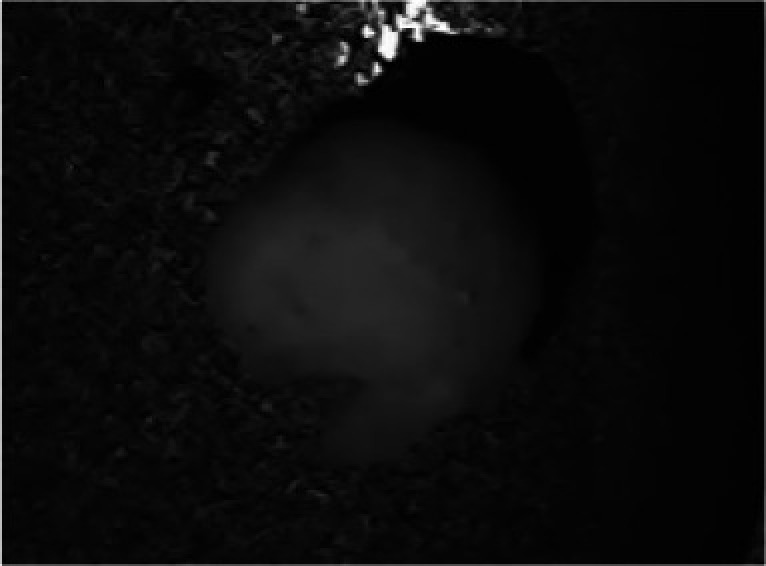	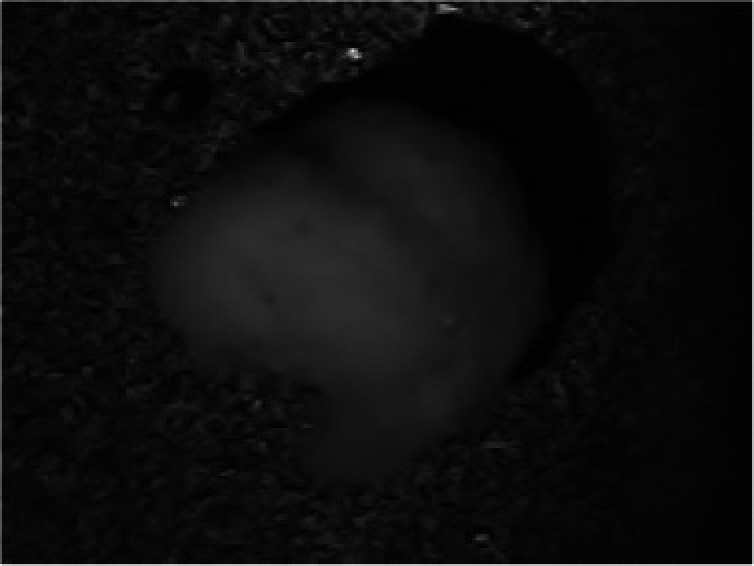	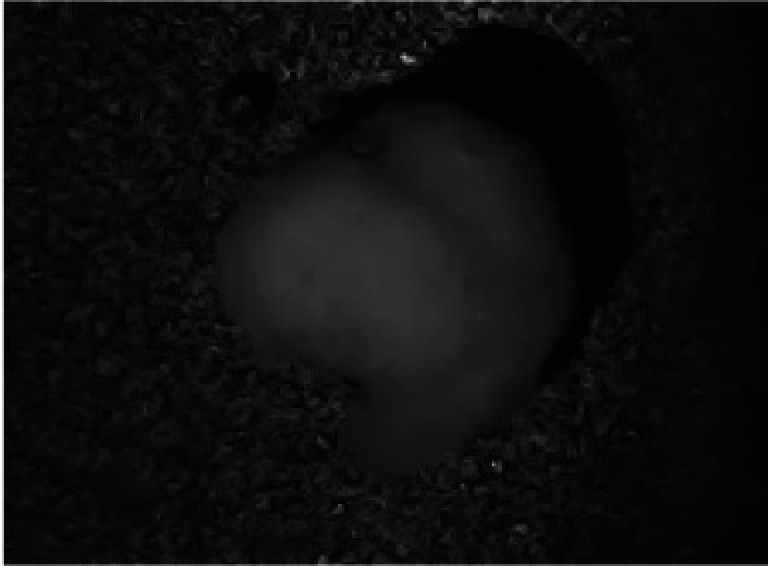

The CP12 pellet disintegration process is depicted in the video in the supplementary material (refer to underlying data). The pellets swelled immediately before exploding and quickly disintegrating. The orphenadrine citrate pellets began to swell and cracks appeared after 120 s, which were easily separated under the oscillating motion of the USP disintegration equipment. The photographs are compatible with the results of the USP disintegration device. When the temperature was increased to 37°C, disintegration caused the fragments to split into smaller fragments. When the temperature was increased to 37°C, disintegration caused the split into tiny fragments. The pseudoephedrine hydrochloride pellets began to explode into several pieces of loosely linked particles within 120 s, which were easily separated by the oscillating motion of the USP disintegration equipment. The photographs were compatible with the results obtained from the USP disintegration device. When the temperature increased to 37°C, the disintegration caused the split into tiny fragments. Although this is not an official USP test, using video capture for disintegration validates Chamsai’s claim of quick disintegration. ^18^

***3.6.5 Drug content***

The drug content of pseudoephedrine hydrochloride and orphenadrine citrate pellets was determined by measuring the absorbance of a specific weight of pellets and calculating the concentration using a linearity equation. As a result, The drug content was API% = 31.8% and 32.1%, respectively, of pellet weight.

***3.6.6 Drug dissolution***

**Figure 10.**
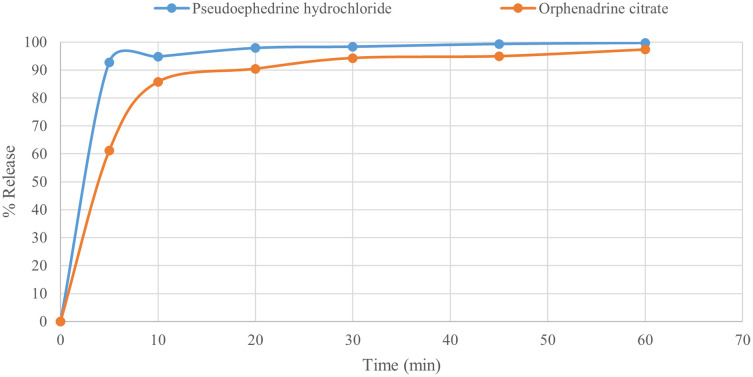
Pseudoephedrine hydrochloride and Orphenadrine citrate dissolution profile.

Dissolution studies in the USP II paddle apparatus revealed that the Pseudoephedrine hydrochloride pellets preparation released more than 95% of its drug in less than 20 minutes ( Figure 10), indicating that the prepared fast-dissolving pellets tend to improve the drug release profile, the disintegration modes reflect the pellets’ dissolution characteristics. This is attributed to the inclusion of the soluble filler mannitol and utilization of the solubilizing power of the hydrophilic polymer PEG 400, resulting in a more porous matrix that facilitates water entry and rapid swelling, complemented by the wicking effect of a combination of disintegrants, which avoids slow diffusion from the insoluble matrix of MCC pellets.

In addition, preparing orphenadrine citrate pellets released more than 90% of the drug in less than 20 min ( Figure 10), indicating that the prepared fast-dissolving pellets tended to improve the drug release profile, and the disintegration modes reflected the pellets’ dissolution characteristics. This is attributed to the inclusion of the soluble filler mannitol and utilization of the solubilizing power of the hydrophilic polymer PEG 400, resulting in a more porous matrix that facilitates water entry and rapid swelling, complemented by the wicking effect of a combination of disintegrants, which avoids the slow diffusion from the insoluble matrix of MCC pellets.

## 4. Conclusion

Extrusion-spheronization is a multistage technique that produces uniformly sized pellets from wet granules. The complex interaction between the equipment, formulation, and process variables, as well as technical knowledge and researcher experience, is critical to the success of these procedures.

Fast-disintegrating pellets were successfully designed and optimized. New formulations of MCC PH 101-based pellets with fast disintegration characteristics have evolved through extrusion and spheronization. Incorporating the soluble filler mannitol, hydrophilic polymer PEG 400, with a super-disintegrant CCS, and PPXL allowed the pellets to explode and disintegrate quickly (10 min). The results revealed that the chosen formula gives pellets a spherical shape, strength, and integrity. The uploading of model drugs and the evaluation of their dissolution were also greatly improved. Fast dissolution of freely soluble drugs, such as pseudoephedrine hydrochloride, and sparingly soluble drugs, such as orphenadrine citrate, was achieved due to pellet disintegration (>90% drug release in 20 min). These findings indicate that disintegrating MCC pellets is useful for improving drug dissolution.

Final pellet evaluation confirmed production pellets that have a high process yield (70%–80%), good pellet sphericity (<AR 1.2), low friability (<1%), and quick disintegration (less than 10 min).

Multi-particulate systems are one of the best dosage forms for children, especially those from preschool years and above, whereas oral dispersible pellets could expand their use to younger children, such as infants and toddlers. Pellets are being investigated for various applications, including immediate and modified release of drugs, implants, orally dispersible preparations, effervescent medicines, and solid dispersions. Established APIs can be reformed into pellets by exploiting their inherent properties and flexibility.

## Declarations

### Ethics approval and consent to participate

Not applicable.

### Consent for publication

Not applicable.

## Data Availability

The data are associated with this article are available on Figshare. The data concerning the dissolution of Pseudoephedrine hydrochloride and Orphenadrine citrate are available on the following link: https://doi.org/10.6084/m9.figshare.29098787 ^28^ Data are available under the terms of the Creative Commons Zero “No rights reserved” license (CC0).
