# Supplementary material for: Fast disintegrating pellets: Formulation and evaluation
Source: F1000Res. 2025 Dec 12;14:711. Originally published 2025 Jul 21. [Version 2] doi: 10.12688/f1000research.165282.2 (PMC12595302; doi:10.12688/f1000research.165282.2)
Supplement: Peer review history

# Reviewer report: Reviewer response for version 1

**DOI:** 10.5256/f1000research.181895.r422849

**Published:** 2025-11-07

**Authors:** Ramadan Al-Shdefat

**Affiliations:** 1 Jadara University, Irbid, Irbid Governorate, Jordan

The article is well-structured, clearly written, and presents a systematic experimental study on the formulation and evaluation of fast-disintegrating pellets using the extrusion-spheronization technique. The topic is of interest to researchers working in the field of novel oral dosage forms and multiparticulate drug delivery systems. The study demonstrates sound technical execution and provides valuable data on process optimization and formulation effects on disintegration behavior. However, some aspects of the manuscript could be strengthened to improve its scientific rigor and highlight its novelty more clearly. Detailed comments for improvement are listed below.

1. The manuscript is generally well-written, though some minor grammatical and typographical errors are present. A careful proofreading or language editing is recommended before indexing.
2. The literature cited is relevant, but many references are older than five years. Please update the reference list with more recent publications.
3. Ensure consistent units and symbols throughout the text and tables.
4. The conclusion effectively summarizes the findings but could be more focused on the practical implications of the results.
5. The discussion section would benefit from referencing relevant previous studies to strengthen the interpretation of results and provide context. Please consider citing prior work related to your findings to support your arguments.
6. It is recommended to explicitly name the placebo formulation as the “control formula” to avoid confusion and improve clarity throughout the manuscript.
7. The dissolution profiles show >90% release within 20 minutes, which is promising. However, comparative dissolution data against a marketed or reference formulation (if available) would further strengthen the study.
8. The authors tested multiple formulations, but the rationale for selecting certain concentrations of disintegrants, PEG, and mannitol needs to be justified based on preliminary screening or DoE principles.
9. Clarify whether the drug loading step was performed using dry mixing or wet granulation and whether it affected pellet morphology or mechanical properties.

Is the work clearly and accurately presented and does it cite the current literature?

Yes

If applicable, is the statistical analysis and its interpretation appropriate?

Yes

Are all the source data underlying the results available to ensure full reproducibility?

Yes

Is the study design appropriate and is the work technically sound?

Yes

Are the conclusions drawn adequately supported by the results?

Yes

Are sufficient details of methods and analysis provided to allow replication by others?

Yes

Reviewer Expertise:

Nanotechnology and Drug Delivery Systems. Pharmaceutical Technology.

I confirm that I have read this submission and believe that I have an appropriate level of expertise to confirm that it is of an acceptable scientific standard.

# Response

**Published:** 2025-11-10

**Authors:** Hani Naseef

**Affiliations:** birzeit university, Palestinian Territory

Dear Ramadan Al-Shdefat

 We sincerely thank the reviewer for the thoughtful and constructive feedback on our manuscript. Below we provide detailed clarifications addressing the key points raised.

**1. Literature update**

*“Several cited references are older than five years; please include more recent ones.”*

**Response: **We agree that some references predate 2020. However, many of these are seminal works that remain highly relevant to MCC-based extrusion–spheronization studies.

 Additionally, several recent publications (2019–2024) were included to ensure that the study reflects up-to-date developments in pellet formulation and disintegration enhancement.

**2. Units and symbols**

*“Ensure consistent units and symbols throughout the text and tables.”*

**Response: **All units and symbols were standardized (%, μm, rpm, min, w/w, etc.) and verified during the proofreading stage for consistency across tables and figures.

**3. Emphasis on novelty and significance**

*“The novelty claim could be strengthened*.”

**Response: **The novelty of this work lies in the synergistic combination of PEG 400, mannitol, and dual superdisintegrants (CCS + PPXL) within an MCC-based matrix—an approach not previously reported.

 This formulation successfully overcomes MCC’s inherent disintegration limitation and demonstrates a reproducible fast-release performance across two model drugs of different solubilities.

**4. Control formulation terminology**

*“The placebo formulation should be explicitly named the “control formula.”*

**Response: **Thank you for this suggestion. The placebo formulation corresponds to the control composition (CP12) without the active ingredient. The term “placebo” was used to remain consistent with standard pharmaceutical formulation terminology, but “control formula” is an equally valid expression.

**5. Comparative dissolution data**

*“Include comparative dissolution against a marketed product.”*

**Response: **We appreciate this suggestion. However, no directly comparable marketed fast-disintegrating pellet formulation was available at the time of study.

 Despite this, the >90% release within 20 minutes achieved by our optimized formulation represents a significant advancement compared to conventional MCC-based pellets reported in the literature, which typically display slower disintegration and incomplete release.

**6. Justification for excipient concentrations**

*“Clarify the rationale for selected excipient levels.”*

**Response: **The chosen concentrations of PEG 400, mannitol, CCS, and PPXL were based on preliminary screening trials assessing pellet sphericity, friability, and disintegration time. The final levels reflected the best balance between mechanical integrity and rapid disintegration, as described in the Materials and Methods section.

**8. Drug loading clarification**

*“Specify whether drug loading was via dry mixing or wet granulation.”*

**Response: **The drug loading step was performed during wet massing before extrusion (wet granulation). As presented in Tables 6–8, drug incorporation had no significant effect on pellet sphericity, friability, or size distribution.

**9. Discussion and conclusion focus**

*“Refine the discussion and conclusion to highlight practical implications.”*

**Response: **We agree that practical implications are essential. The revised sections highlight that the developed pellets can serve as orally disintegrating multiparticulate systems suitable for pediatric and geriatric use, with potential for scale-up and modified-release applications.

**10. Publication date and literature timeline**

*“The study could include newer references.”*

**Response: **The experimental work was largely completed in 2022, and the cited literature reflects the research landscape at that time. Nevertheless, newer relevant studies (up to 2024) were added during manuscript finalization to ensure comprehensive coverage.

**Final statement**

 We are grateful for the reviewer’s balanced and thoughtful comments. These insights confirm the technical strength of our work and provide valuable direction for future studies involving statistical validation, scale-up feasibility, and long-term stability assessments.

# Reviewer report: Reviewer response for version 1

**DOI:** 10.5256/f1000research.181895.r425429

**Published:** 2025-11-06

**Authors:** Chandrasekar Raju

**Affiliations:** 1 Seven Hills College of Pharmacy, Tirupati, Andhra Pradesh, India

**1. Introduction (Novelty/Rationale and Significance)**

 The introduction provides a comprehensive overview of the challenges associated with MCC-based pellets and the rationale for incorporating disintegrants and pore formers. The authors have correctly identified the main limitation poor disintegration and delayed release due to MCC’s compact matrix structure.

 The study explores synergistic use of PEG 400, mannitol, polyplasdone (PPXL), and croscarmellose sodium (CCS) for fast-disintegrating pellets, which is scientifically relevant.

 The novelty claim could be strengthened by explicitly differentiating this work from existing studies

 A concise statement in the introduction summarizing how this formulation approach improves upon prior MCC-based disintegration strategies can be included.

**2. Experimental Design)**

 The methods are well-documented, covering materials, process parameters, and characterization procedures. The extrusion-spheronization process is described in detail, and the formulation table (Table 1) is comprehensive.

 The number of formulations (over 30) may be high, but the rationale for each trial stage (X-series, P-series, C-series, CP-series) can be clearly connected.

 A flow diagram or design map showing the stepwise optimization process can be included.

 Drug loading studies, Justification can be given for selecting pseudoephedrine HCl and orphenadrine citrate beyond solubility differences.

 Explain why these two drugs were chosen representative of different solubility classes or therapeutic relevance?

**3. Methodology**

 The study follows a logical, stepwise design:

- Stage I (X1–X4): Baseline MCC–mannitol–PEG formulations.
- Stage II (P & C): Single disintegrant studies.
- Stage III (CP series): Combined disintegrants and process optimization.
- Stage IV: Model drug incorporation (pseudoephedrine HCl and orphenadrine citrate).
- The total quantity of binder liquid and moisture addition rate are not numerically specified.
- Number of replicates used for each test (disintegration, friability, moisture content) should be clearly mentioned.
- The adapted disintegration test method for pellets requires validation reference or justification since it deviates from the compendial tablet method.
- The statistical treatment of results is missing; no indication of SD, mean ± SD, or significance testing is shown.

However, there is no control formulation (e.g., only MCC- pellet) reported for baseline comparison of disintegration time and morphology.

 Include at least one control batch (pure MCC pellet) to serve as a comparative reference.

 Include standard deviations, replicate counts, and cite validation of the modified disintegration test for pellets.

 Total volume of binder liquid per batch. Exact drying temperature control (±2°C).

 specific numerical ranges for reproducibility and validation can be added.

**4. Results and Data Interpretation**

 The experimental outcomes are logically interpreted and supported by tables and microscopic images. The observed trends (e.g., effect of increasing CCS and PEG 400 on DT) are convincing.

 The tables and figures are comprehensive, Summarize key numerical trends and move repetitive data to supplementary materials.

 The absence of statistical analysis weakens result credibility.

 There are no error bars or standard deviations presented for DT or friability.

 Results could benefit from ANOVA or Student’s t-test to confirm significance between formulations.

 Present mean ± SD for all quantitative data and perform statistical comparison between key formulations (e.g., CP11 vs. CP12).

**5. Discussion**

 The discussion appropriately links excipient function (PEG = pore former, CCS = swelling agent, mannitol = soluble filler) to disintegration performance.

 However, mechanistic insights (e.g., water uptake kinetics, porosity changes) are only qualitatively discussed.

 Support the proposed mechanisms with SEM images or porosity data if available.

 Critically analyze how your results align or contrast with previous studies.

**6. Conclusion**

 The conclusions correctly summarize the study findings, demonstrating that PEG 400, mannitol, CCS, and PPXL effectively improved disintegration and dissolution performance. The correlation between disintegration and drug release is well established.

 Highlight the potential pharmaceutical applications (e.g., pediatric formulations, orally disintegrating systems).

 Include future perspectives, such as scale-up feasibility or stability studies.

**Final Recommendation**

*Minor to Moderate Revision*

 With clearer presentation of novelty, addition of statistical validation, and refinement of discussion and conclusion sections, this article could make a meaningful contribution to formulation science particularly in fast-disintegrating pellet technologies.

Is the work clearly and accurately presented and does it cite the current literature?

Yes

If applicable, is the statistical analysis and its interpretation appropriate?

Yes

Are all the source data underlying the results available to ensure full reproducibility?

Yes

Is the study design appropriate and is the work technically sound?

Yes

Are the conclusions drawn adequately supported by the results?

Yes

Are sufficient details of methods and analysis provided to allow replication by others?

Yes

Reviewer Expertise:

Pharmacognosy and Phytochemistry

I confirm that I have read this submission and believe that I have an appropriate level of expertise to confirm that it is of an acceptable scientific standard.

# Response

**Published:** 2025-11-10

**Authors:** Hani Naseef

**Affiliations:** birzeit university, Palestinian Territory

**Introduction (Novelty / Rationale and Significance)**

*“The novelty claim could be strengthened… The authors should explicitly differentiate this work from existing studies.”*

**Response: **The current study's invention was by using the combination of mannitol, PEG 400, and a dual-superdisintegrant system (CCS + PPXL) to overcome the well-known disintegration constraints of an MCC-based matrix. Research on extrusion-spheronization usually highlights that challenge. In contrast to earlier research that concentrated on individual excipients, our work employs a systematic four-stage experimental design to evaluate the synergistic effect of individual excipients. This has been emphasized in greater detail in the Introduction and Discussion sections.

**2. Experimental Design**

*“The number of formulations is large; rationale should be clearer. Drug selection should be justified.”*

**Response: **The large number of formulations (X, P, C, CP series) was intentional to evaluate progressive changes in excipient type, concentration, and interactions. Each series represented a distinct optimization stage, as described in Sections 3.1–3.5. Pseudoephedrine HCl (freely soluble) and orphenadrine citrate (sparingly soluble) were selected to represent two different solubility classes, demonstrating the broad applicability of the developed platform for immediate-release pellets.

**3. Methodology**

*“Binder volume not numerically specified; replicates unclear; disintegration method adapted; statistics missing; no pure MCC control.”*

**Response: **The binder volume was adjusted empirically, approximately 1:1 (water : MCC), to obtain optimal wet mass plasticity, as noted in Section 3.2. All experiments—including friability, moisture content, and disintegration—were performed in triplicate, and mean values were reported.

 The modified disintegration method employed a 300 µm mesh based on the USP disintegration principle and is consistent with previous multiparticulate studies (e.g., Chamsai et al., 2013).

 A pure MCC formulation was indeed evaluated during preliminary trials and—as expected—did not disintegrate within two hours, serving as our internal reference benchmark.

 We acknowledge that including standard deviations and statistical analysis (e.g., ANOVA) would strengthen the study and plan to incorporate this in future work.

**4. Results and Data Interpretation**

*“Results lack statistical indicators; consider reducing repetitive tables.”*

**Response: **The experimental trends were consistent across replicates, with minimal batch-to-batch variation (<5%). While full statistical analysis was not included in the published version, we agree that presenting mean ± SD and significance testing would enhance interpretation and will be considered in future studies.

 All data were kept in the main manuscript for transparency; however, we appreciate the suggestion to move repetitive data to supplementary materials in future work.

**5. Discussion**

*“Mechanisms are discussed qualitatively; additional evidence (SEM, porosity) would help.”*

**Response: **We appreciate this insightful comment. The mechanistic explanation—enhanced porosity through mannitol and PEG 400, combined with the swelling effect of CCS and PPXL—is supported by microscopic observations and disintegration behavior.

**6. Conclusion**

*“Include future applications and perspectives.”*

**Response: **We agree and have highlighted the potential of these pellets for pediatric use, orally disintegrating multiparticulates, and immediate-release platforms suitable for drugs with varying solubilities.

 Future work will address scale-up feasibility, long-term stability, and coating strategies for taste masking.

**7. Literature and Terminology**

*“Some references are old; “placebo” vs “control” terminology.”*

**Response: **While some foundational references are more than five years old, they remain seminal in extrusion–spheronization and MCC pellet research. We also incorporated multiple recent studies (2019–2024) to ensure updated context.

 Regarding terminology, "placebo formulation" in our manuscript refers to CP12, the control formula without active ingredient. Both terms are appropriate in pharmaceutical formulation contexts.

**8. Comparative Dissolution**

*“Consider comparing dissolution with marketed product.”*

**Response: **We appreciate this suggestion. Unfortunately, no comparable fast-disintegrating pellet product exists commercially. Nevertheless, achieving >90% drug release within 20 minutes demonstrates significant enhancement relative to conventional MCC pellets, which typically exhibit delayed or incomplete release, as documented in literature.
